# Serum sCD163 as a potential biomarker for predicting poor functional outcome at 3 months in acute ischemic stroke without reperfusion: a prospective study

**DOI:** 10.3389/fimmu.2026.1914293

**Published:** 2026-08-11

**Authors:** Meijuan Yan, Lihui Shao, Zhining Li, Chunfeng Liu

**Affiliations:** 1 Department of Neurology, the Second Affiliated Hospital of Soochow University, Suzhou, China; 2 Department of Neurology, the Affiliated Municipal Hospital of Xuzhou Medical University, Xuzhou, China; 3 Department of Rehabilitation, Xinyi Hospital of Traditional Chinese Medicine, Xuzhou, China; 4 Department of Neurology, the Second Affiliated Hospital of Xuzhou Medical University, Xuzhou, China

**Keywords:** acute ischemic stroke, external validation, functional outcome, neuroinflammation, prognostic model, soluble CD163

## Abstract

**Introduction:**

Neuroinflammation plays a critical role in the prognosis of acute ischemic stroke (AIS). Soluble CD163 (sCD163), a marker of monocyte/macrophage activation, has been linked to stroke severity, but its independent prognostic value and clinical utility remain uncertain. This study aimed to evaluate whether serum sCD163 measured within 24 hours of admission predicts 3_month poor functional outcome in AIS patients without reperfusion therapy, and to construct and externally validate a prognostic model incorporating sCD163.

**Methods:**

This prospective, dual_cohort study enrolled 664 first_ever AIS patients (training cohort, 2021 -2023) and 300 patients from an independent center (external validation cohort, 2024). All patients did not receive intravenous thrombolysis or mechanical thrombectomy. Serum sCD163 levels were measured at admission by ELISA. The primary outcome was poor functional outcome at 3 months, defined as modified Rankin Scale (mRS) > 2. Multivariable logistic regression with LASSO variable selection was used to identify independent predictors. Model performance was assessed by discrimination (AUC), calibration (Hosmer_Lemeshow test), and decision curve analysis (DCA), with internal bootstrap validation and external validation.

**Results:**

Poor outcome occurred in 34.1% (226/664) of the training cohort and 34.0% (102/300) of the validation cohort. Serum sCD163 levels were significantly higher in poor_outcome patients (820 ± 110 ng/mL vs. 650 ± 80 ng/mL, P < 0.001). The optimal cutoff was 742.5 ng/mL. After multivariable adjustment, high sCD163 (≥ 742.5 ng/mL) was an independent predictor of poor outcome (adjusted OR = 7.52, 95% CI: 4.42 -12.78, P < 0.001), along with baseline NIHSS score, DWI infarct volume, and atrial fibrillation. The combined model achieved an internal bootstrap_validated AUC of 0.877 (95% CI: 0.829 -0.926) and an external validation AUC of 0.867 (95% CI: 0.815 -0.921), with good calibration (Hosmer_Lemeshow P = 0.868 and 0.680, respectively) and superior net clinical benefit on DCA. Adding sCD163 significantly improved model performance over clinical predictors alone (ΔAUC = 0.118, P < 0.001).

**Discussion:**

Serum sCD163 within 24 hours of admission is a robust, independent biomarker for predicting 3_month poor functional outcome in AIS patients without reperfusion. The prognostic model combining sCD163 with traditional clinical and imaging variables demonstrates excellent discrimination, calibration, and clinical utility in an independent external cohort. Our findings support the early measurement of sCD163 for individualized risk stratification, though further mechanistic and multi_center studies are needed to confirm generalizability and causality.

## Introduction

Acute ischemic stroke (AIS) is one of the leading causes of death and long-term neurological disability worldwide (1). In China, the incidence, prevalence, and disease burden of stroke remain among the highest globally, imposing heavy pressure on patients’ families and the healthcare system (2). The long-term functional prognosis of AIS patients is significantly heterogeneous, with approximately 30–40% of patients developing moderate-to-severe neurological deficits at 3 months after onset, severely affecting quality of life and social participation (3, 4). Therefore, early and accurate identification of patients at risk of poor prognosis is of critical importance for guiding individualized treatment, rehabilitation strategies, and physician-patient communication.

Currently, core indicators for prognostic assessment of AIS mainly include baseline demographic and vascular risk factors, the National Institutes of Health Stroke Scale (NIHSS) score, and neuroimaging parameters such as diffusion-weighted imaging (DWI) infarct volume and site of vessel occlusion (5, 6). However, these traditional indicators have obvious limitations: the NIHSS score is subject to inter-rater variability, DWI volume measurement requires specialized equipment and expertise, and the predictive performance of any single indicator is limited, making it difficult to fully capture the patient’s pathophysiological status and long-term prognosis (7, 8). Therefore, identifying objective, quantifiable, and easily measurable serum biomarkers and combining them with traditional clinical and imaging indices to build accurate prognostic prediction models is a major research focus in the stroke field.

CD163 is a scavenger receptor mainly expressed on the surface of monocytes/macrophages and belongs to the cysteine-rich superfamily. Its core physiological functions include recognition and clearance of the hemoglobin-haptoglobin complex, as well as participation in the regulation of inflammatory responses, maintenance of immune homeostasis, and tissue repair (9, 10). Under pathological conditions, CD163 can be shed from the macrophage surface by proteolytic cleavage, releasing soluble CD163 (sCD163) into the peripheral blood. Serum sCD163 levels directly reflect the activation status of macrophages and the intensity of inflammation (11, 12). In recent years, increasing evidence has confirmed that sCD163 plays a key role in various inflammation-related diseases, including cardiovascular disease, autoimmune disorders, and neurodegenerative diseases (13–15).

In the field of ischemic stroke, small-sample studies have found that peripheral blood sCD163 levels are significantly elevated in AIS patients compared with healthy controls, and that sCD163 levels correlate positively with infarct volume and the degree of neurological deficit (16, 17). Nevertheless, the independent prognostic value of sCD163 for long-term functional outcomes in AIS remains controversial. Most studies have limited sample sizes, insufficient adjustment for multiple confounders, and lack independent external validation, making it difficult to establish sCD163 as an independent risk factor for poor prognosis (18, 19). Moreover, studies that combine sCD163 with traditional clinical and imaging indices to construct AIS prognostic models are still lacking, and the clinical utility of this biomarker requires further systematic validation.

This prospective dual-cohort study enrolled 664 first-ever AIS patients as a training cohort and 300 patients from an independent center as a validation cohort. All enrolled patients did not receive reperfusion therapies (intravenous thrombolysis or mechanical thrombectomy). The primary aims were: (1) to investigate the association between serum sCD163 levels measured within 24 hours of admission and poor functional outcome at 3 months; (2) to determine whether sCD163 is an independent predictor of poor 3-month outcome; and (3) to construct a prediction model combining sCD163 with traditional clinical and imaging predictors (NIHSS score, DWI infarct volume, and atrial fibrillation) and to systematically validate its performance in an independent cohort. The results are expected to provide a novel, reliable serum biomarker for early prognostic assessment of AIS and a high-quality theoretical basis for individualized treatment decisions.

## Methods

### Study population

Consecutive patients with acute ischemic stroke (AIS) were prospectively enrolled from two independent centers between January 2021 and December 2024. All participants underwent comprehensive clinical evaluation, including detailed medical history collection, laboratory tests, neurological examination, cranial computed tomography (CT), and magnetic resonance imaging (MRI). According to the diagnostic criteria established by the American Heart Association/American Stroke Association, a total of 664 eligible patients admitted to the Affiliated Municipal Hospital of Xuzhou Medical University from January 2021 to December 2023 were initially screened and constituted the training cohort. Subsequently, 300 eligible patients admitted to the Second Affiliated Hospital of Xuzhou Medical University, a separate institution, from January 2024 to December 2024 were enrolled as an independent validation cohort. The two cohorts were temporally and institutionally independent, with no overlap in recruitment periods or patient populations. All enrolled patients were newly diagnosed with first-ever AIS and did not receive any reperfusion therapy, including intravenous thrombolysis or mechanical thrombectomy. Peripheral blood samples were collected within 24 hours of admission.

The study protocol was approved by the Institutional Ethics Committee of the Affiliated Municipal Hospital of Xuzhou Medical University (Approval No. XZYY2021-KL01-038). The external validation cohort was collected following approval from the Institutional Ethics Committee of the Second Affiliated Hospital of Xuzhou Medical University (Approval No. KZYY2024-KL05-023). All procedures were performed in accordance with the ethical standards of the institutional research committees and the 1964 Helsinki Declaration and its later amendments. Written informed consent was obtained from all participants or their legal representatives prior to enrollment.

Inclusion criteria were: (1) age 18–85 years; (2) admission within 24 hours of symptom onset; (3) confirmed AIS by cranial CT or MRI; (4) admission National Institutes of Health Stroke Scale (NIHSS) score between 4 and 22; (5) did not receive any reperfusion therapy; (6) first-ever stroke or pre-stroke modified Rankin Scale (mRS) score ≤ 1.

Exclusion criteria were: (1) pre-stroke mRS ≥ 2; (2) definite autoimmune diseases or immunodeficiency; (3) use of immunosuppressants, glucocorticoids, or immunomodulators within the past 3 months; (4) blood transfusion or intravenous immunoglobulin within the past 3 months; (5) active infection or fever (body temperature ≥ 38.0 °C) at admission; (6) severe hepatic or renal dysfunction; (7) active malignancy; (8) pregnancy or lactation; (9) death within 48 hours of admission.

### Clinical data collection

Demographic characteristics (age, sex, body mass index [BMI]), lifestyle factors (smoking, alcohol consumption), vascular risk factors (hypertension, diabetes mellitus, hypercholesterolemia, atrial fibrillation, family history of stroke), and medication history (antiplatelet and anticoagulant drug use) were collected at admission through standardized interviews and electronic medical record review. Stroke severity was assessed using the NIHSS at admission, daily for the first 7 days, and at discharge. MRI was performed within 24 hours of admission, prior to any intervention. DWI infarct volume was measured on baseline cranial MRI using a semi-automated segmentation method implemented in ITK-SNAP (version 3.8, www.itksnap.org). Infarct location (anterior vs. posterior circulation), distribution of culprit vessels (internal carotid artery, middle cerebral artery, or basilar artery), and vessel stenosis degree (normal, stenosis, or occlusion) were also recorded based on baseline MRI and MR angiography findings. Onset-to-admission time was recorded for each patient.

Fasting venous blood samples were collected within 24 hours of admission. Laboratory parameters, including random blood glucose, white blood cell count, neutrophil percentage, platelet count, fibrinogen, and international normalized ratio (INR), were measured using standard automated laboratory techniques at the central clinical laboratory of each participating center. All laboratory measurements were performed as part of routine clinical care, and the technicians performing the assays were blinded to patient outcomes.

Stroke etiological classification was determined according to the Trial of Org 10172 in Acute Stroke Treatment (TOAST) criteria by two independent board-certified neurologists; disagreements were resolved by consensus with a third senior neurologist.

Outcome definition: The primary endpoint was functional outcome at 3 months, assessed using the modified Rankin Scale (mRS). A good outcome was defined as mRS ≤ 2 (no or mild disability, able to live independently), while a poor outcome was defined as mRS > 2 (moderate to severe disability, bedridden, dependence on others, or death). The mRS assessment was performed at 90 ± 7 days after stroke onset by trained neurologists who were blinded to all laboratory data, clinical information, and baseline characteristics of the patients. All evaluators had at least 3 years of clinical experience in stroke care and had undergone standardized training in the administration of the modified Rankin Scale, including the use of a structured questionnaire and adherence to the standardized mRS interview protocol. Inter-rater reliability was assessed in a subset of patients (n = 30), yielding a weighted κ coefficient of 0.86 (95% CI: 0.78–0.94), indicating excellent agreement. Assessments were conducted via face-to-face interviews at the 3-month outpatient follow-up visit or, for patients unable to attend in person, via standardized telephone interviews using a validated structured mRS questionnaire.

### Serum sCD163 measurement

Peripheral venous blood was collected within 24 hours of admission in plain tubes without anticoagulant. Samples were allowed to clot at room temperature for 30 minutes and centrifuged at 3000 rpm for 10 minutes at 4 °C. Serum was aliquoted and stored at −80 °C until batch analysis for sCD163 and cytokine measurements. Serum sCD163 levels were measured in duplicate using a commercial enzyme-linked immunosorbent assay (ELISA) kit (R&D Systems, Minneapolis, MN, USA) according to the manufacturer’s instructions. All ELISA measurements were performed within 6 months of sample collection. The intra-assay and inter-assay coefficients of variation were <8% and <10%, respectively. Laboratory technicians performing the assays were blinded to all clinical and outcome data.

### Statistical analysis

SPSS 26.0 and R 3.6.0 software were used for statistical analysis. Normally distributed continuous variables were expressed as mean ± standard deviation and compared using the independent samples t-test; non-normally distributed variables were presented as median (interquartile range) and compared using the Mann-Whitney U test. Categorical variables were described as counts (percentages) and compared using the χ² test.

#### Missing data

We assessed the pattern of missing data using the md.pattern() function from the mice package in R to determine the missing data mechanism. We used multiple imputation to handle missing data, performing 10 imputations based on standard recommendations under the confirmed MAR mechanism. The Multiple Imputation by Chained Equations (MICE) approach was employed using the mice package in R. Continuous variables were imputed with predictive mean matching, categorical variables were imputed with logistic regression, and multinomial variables were imputed with polytomous regression (multinomial logistic regression). All relevant covariates, including predictors, the outcome variable, and other variables not included in the predictive model, were included in the imputation model to capture the relationships between variables.

#### Determination of the optimal cutoff value for serum sCD163

The receiver operating characteristic (ROC) curve was plotted to evaluate the discriminative ability of serum sCD163 for predicting 3-month poor functional outcome. The optimal cutoff value was determined using the Youden index to maximize sensitivity and specificity.

#### Predictor selection and model development

Candidate predictors were pre-specified based on clinical relevance and prior literature. Variable selection was performed using least absolute shrinkage and selection operator (LASSO) regression with 10-fold cross-validation, employing the “1-standard error” (1-SE) rule to select the optimal penalty parameter λ. Variables with non-zero coefficients at the optimal λ were selected for further analysis. The selected variables were subsequently entered into a multivariable logistic regression model using a forced entry method to identify independent predictors of 3-month poor functional outcome.

#### Collinearity and interaction diagnostics

Collinearity diagnostics were performed using variance inflation factor (VIF), with VIF < 5 indicating no significant multicollinearity. Interaction effects were tested by introducing interaction terms into the regression model, with *P* for interaction > 0.1 indicating no substantial interactions.

#### Internal and external validation

For internal validation, bootstrap resampling with 1,000 iterations was performed to assess model optimism and provide optimism-corrected performance estimates (AUC, R², and Brier score) in the training cohort. Calibration intercept and slope were also calculated from the bootstrap samples. For external validation, the original model coefficients were directly applied to the independent validation cohort (n = 300) without recalibration, as recommended by the TRIPOD guidelines. The same performance metrics (AUC, calibration plot, Hosmer-Lemeshow test, and DCA) were evaluated in the validation cohort.

#### Incremental predictive value

To evaluate the additional prognostic information provided by sCD163 beyond traditional clinical and imaging predictors, we compared the clinical model (baseline NIHSS + DWI infarct volume + atrial fibrillation) with the full model (clinical model + sCD163). Model discrimination was compared using the DeLong test to assess the statistical significance of the AUC difference. Model fit improvement was evaluated using the likelihood ratio test, comparing the −2 log-likelihood values between the nested models. Additionally, we calculated the net reclassification improvement (NRI) and integrated discrimination improvement (IDI) to quantify the improvement in risk reclassification and overall discrimination after adding sCD163 to the clinical model. *P* values for NRI and IDI were derived from bootstrap resampling with 1,000 iterations.

#### Sensitivity analyses

To assess the robustness of our findings, two sensitivity analyses were conducted: (1) including patients who died within 48 hours of admission (n = 10, with 3-month mRS assigned a value of 6 and missing sCD163 values handled by multiple imputation) to address potential survivor bias; and (2) treating sCD163 as a continuous variable (per 10 ng/mL increase) to address the potential overoptimism associated with ROC-derived dichotomization.

A two-tailed *P* < 0.05 was considered statistically significant for independent predictors in the multivariable model, except for interaction tests where *P* > 0.1 was considered indicative of no significant interaction.

## Results

### Study population and patient flow

**Figure 1:**
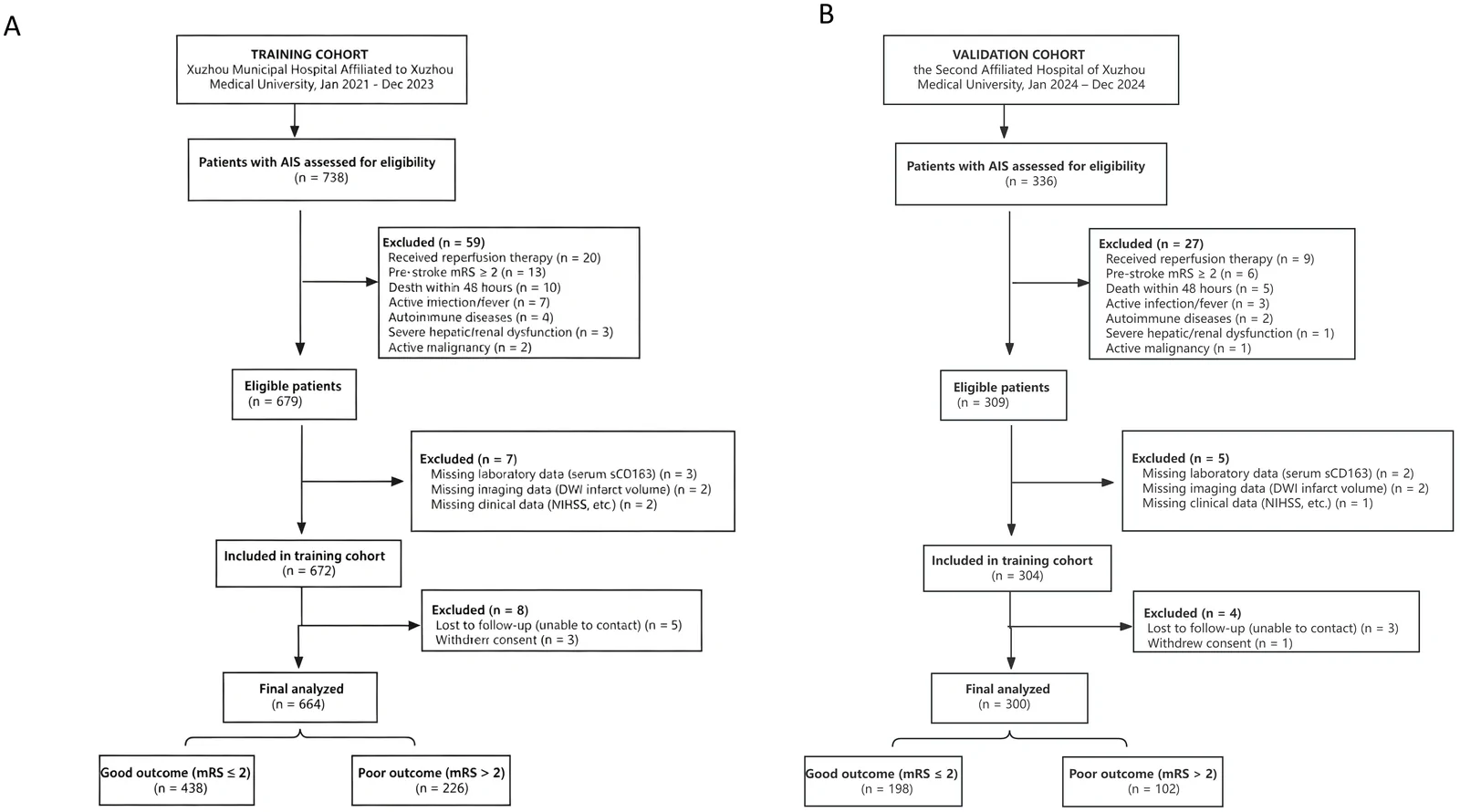
Study flow diagram. **(A)** Training cohort. Between January 2021 and December 2023, 738 patients with acute ischemic stroke (AIS) were assessed for eligibility at the Affiliated Municipal Hospital of Xuzhou Medical University. **(B)** Validation cohort. Between January 2024 and December 2024, 336 patients with AIS were assessed for eligibility at the Second Affiliated Hospital of Xuzhou Medical University.

A total of 1,074 patients with acute ischemic stroke were screened from two independent centers between January 2021 and December 2024 (**Figure 1**). In the training cohort (the Affiliated Municipal Hospital of Xuzhou Medical University, January 2021–December 2023, n = 738 screened), 59 patients were excluded based on the exclusion criteria, 7 had missing data, and 8 were lost to follow-up (5 lost to contact, 3 withdrew consent), leaving 664 patients for final analysis (438 good outcomes, 226 poor outcomes). In the validation cohort (the Second Affiliated Hospital of Xuzhou Medical University, January 2024 – December 2024, n = 336 screened), 27 patients were excluded based on the exclusion criteria, 5 had missing data, and 4 were lost to follow-up (3 lost to contact, 1 withdrew consent), leaving 300 patients for final analysis (198 good outcomes, 102 poor outcomes). The two cohorts were temporally and institutionally independent, with no overlap in recruitment periods or patient populations. All remaining patients completed the 3-month follow-up, and no deaths occurred during the follow-up period.

### Baseline characteristics between the training and validation cohorts

In the current dataset, there were 17 variables with missing data, comprising a total of 385 missing values, which accounts for approximately 1.48% of the total data points. The missing values were distributed across multiple variables, as detailed in **Supplementary Table 7**. As shown in **Table 1**, there were no significant differences in age, sex, smoking, alcohol consumption, BMI, hypertension, hypercholesterolemia, diabetes, atrial fibrillation, family history of stroke, antiplatelet drug use, anticoagulant drug use, baseline NIHSS, DWI infarct volume, random blood glucose, white blood cell count, neutrophil percentage, platelet count, fibrinogen, INR, infarct location, distribution of culprit vessels, vessel stenosis degree, onset-to-admission time, TOAST classification, and serum sCD163 levels between the training cohort (n = 664) and the validation cohort (n = 300) (all *P* > 0.05). In addition to the *P* values, we calculated standardized mean differences (SMD) to quantify the magnitude of differences between the two cohorts. All SMD values were below 0.1 (range: 0.017 to 0.097 for most variables; the largest SMD was 0.139 for neutrophil percentage, still indicating acceptable balance), confirming excellent comparability between the training and validation cohorts (**Supplementary Table 10**).

**Table 1:** Baseline characteristics of the training and validation cohorts.

Variable	Training cohort (n=664)	Validation cohort (n=300)	Statistic (t/χ²/Z)	*P* value
Demographic characteristics				
Age (years, mean ± SD)	65.6 ± 11.1	65.3 ± 11.5	t = 0.41	0.682
Male, n (%)	430 (64.8)	192 (64.0)	χ² = 0.06	0.810
Smoking, n (%)	253 (38.1)	109 (36.3)	χ² = 0.29	0.590
BMI [M (Q1, Q3)]	24.3 (22.6, 26.4)	24.2 (22.5, 26.3)	Z = 0.55	0.582
Alcohol consumption, n (%)	162 (24.4)	78 (26.0)	χ² = 0.30	0.584
Vascular risk factors and comorbidities				
Hypertension, n (%)	416 (62.7)	191 (63.7)	χ² = 0.09	0.765
Hypercholesterolemia, n (%)	273 (41.1)	127 (42.3)	χ² = 0.13	0.718
Diabetes, n (%)	188 (28.3)	80 (26.7)	χ² = 0.28	0.597
Atrial fibrillation, n (%)	167 (25.2)	71 (23.7)	χ² = 0.25	0.621
Family history of stroke, n (%)	89 (13.4)	44 (14.7)	χ² = 0.29	0.590
Medication history				
Antiplatelet drug use, n (%)	302 (45.5)	130 (43.3)	χ² = 0.40	0.527
Anticoagulant drug use, n (%)	103 (15.5)	50 (16.7)	χ² = 0.21	0.647
Clinical severity and imaging findings				
Baseline NIHSS [M (Q1, Q3)]	8 (6, 12)	8 (6, 12)	Z = 0.38	0.704
DWI infarct volume (ml, mean ± SD)	19.6 ± 12.5	19.1 ± 12.8	t = 0.57	0.569
Random blood glucose (mmol/L, mean ± SD)	8.03 ± 2.82	8.31 ± 2.98	t = 1.38	0.168
White blood cell count (×10^9^/L, mean ± SD)	8.40 ± 3.00	8.62 ± 3.18	t = 1.03	0.304
Neutrophil percentage (%, mean ± SD)	0.712 ± 0.11	0.728 ± 0.12	t = 1.66	0.097
Platelet count (×10^9^/L, mean ± SD)	218 ± 56	221 ± 59	t = 0.72	0.471
Fibrinogen (g/L, mean ± SD)	3.26 ± 0.79	3.23 ± 0.77	t = 0.54	0.587
INR (mean ± SD)	1.03 ± 0.07	1.04 ± 0.08	t = 1.73	0.084
Infarct location, n (%)			χ² = 2.21	0.137
– Anterior circulation	379 (57.1)	182 (60.7)		
– Posterior circulation	285 (42.9)	118 (39.3)		
Distribution of culprit vessels, n (%)			χ² = 1.89	0.389
– Internal carotid artery	56 (8.4)	21 (7.0)		
– Middle cerebral artery	344 (51.8)	162 (54.0)		
– Basilar artery	264 (39.8)	117 (39.0)		
Vessel stenosis degree, n (%)			χ² = 2.99	0.225
– Normal	96 (14.5)	39 (13.0)		
– Stenosis	214 (32.2)	94 (31.3)		
– Occlusion	354 (53.3)	167 (55.7)		
Onset-to-admission time (h) [M (Q1, Q3)]	8.0 (4.5, 13.5)	8.0 (4.5, 13.5)	Z = 0.21	0.834
TOAST classification, n (%)			χ² = 4.02	0.408
– Large artery atherosclerosis	256 (38.6)	124 (41.3)		
– Cardioembolism	182 (27.4)	87 (29.0)		
– Small vessel occlusion	148 (22.3)	62 (20.7)		
– Other determined etiology	48 (7.2)	14 (4.7)		
– Undetermined etiology	30 (4.5)	13 (4.3)		
Laboratory biomarker				
sCD163 (ng/mL, mean ± SD)	709 ± 108	705 ± 110	t = 0.54	0.589

### Baseline characteristics stratified by 3-month functional outcome

Among the 664 patients in the training cohort, 438 (65.9%) achieved a favorable 3-month functional outcome (mRS ≤ 2), whereas 226 (34.1%) had a poor functional outcome (mRS > 2). As summarized in **Supplementary Table 1**, there were no significant intergroup differences in age, sex, smoking, alcohol consumption, BMI, hypertension, hypercholesterolemia, diabetes, family history of stroke, prior antiplatelet or anticoagulant medication, onset-to-admission time, infarct location, distribution of culprit vessels, vessel stenosis degree, or TOAST subtypes (all *P* > 0.05). In contrast, patients with an unfavorable prognosis exhibited a significantly higher proportion of atrial fibrillation (31.4% vs. 21.9%, *P* = 0.014), higher median baseline NIHSS scores [12 (9–17) vs. 7 (5–10), *P* = 0.007], larger DWI infarct volumes (28.5 ± 14.0 mL vs. 15.2 ± 8.5 mL, *P* < 0.001), and markedly elevated serum sCD163 levels (820 ± 110 ng/mL vs. 650 ± 80 ng/mL, *P* < 0.001).

Among the 300 patients in the validation cohort, 198 (66.0%) achieved a favorable outcome (mRS ≤ 2), while 102 (34.0%) had a poor outcome (mRS > 2). Similar to the training cohort, there were no significant intergroup differences in age, sex, smoking, alcohol consumption, BMI, hypertension, hypercholesterolemia, diabetes, family history of stroke, prior antiplatelet or anticoagulant medication, onset-to-admission time, infarct location, distribution of culprit vessels, vessel stenosis degree, or TOAST subtypes (all *P* > 0.05). In contrast, patients with an unfavorable prognosis exhibited a significantly higher proportion of atrial fibrillation (32.4% vs. 19.2%, *P* = 0.013), higher median baseline NIHSS scores [12 (8–16) vs. 7 (5–10), *P* = 0.012], larger DWI infarct volumes (27.0 ± 14.2 mL vs. 15.0 ± 8.8 mL, *P* < 0.001), and markedly elevated serum sCD163 levels (797 ± 115 ng/mL vs. 658 ± 78 ng/mL, *P* < 0.001). These findings were consistent with those observed in the training cohort, further supporting the robust association of these factors with poor functional outcome in AIS patients (**Supplementary Table 2**).

### Stratified analysis of serum sCD163 levels

**Figure 2:**
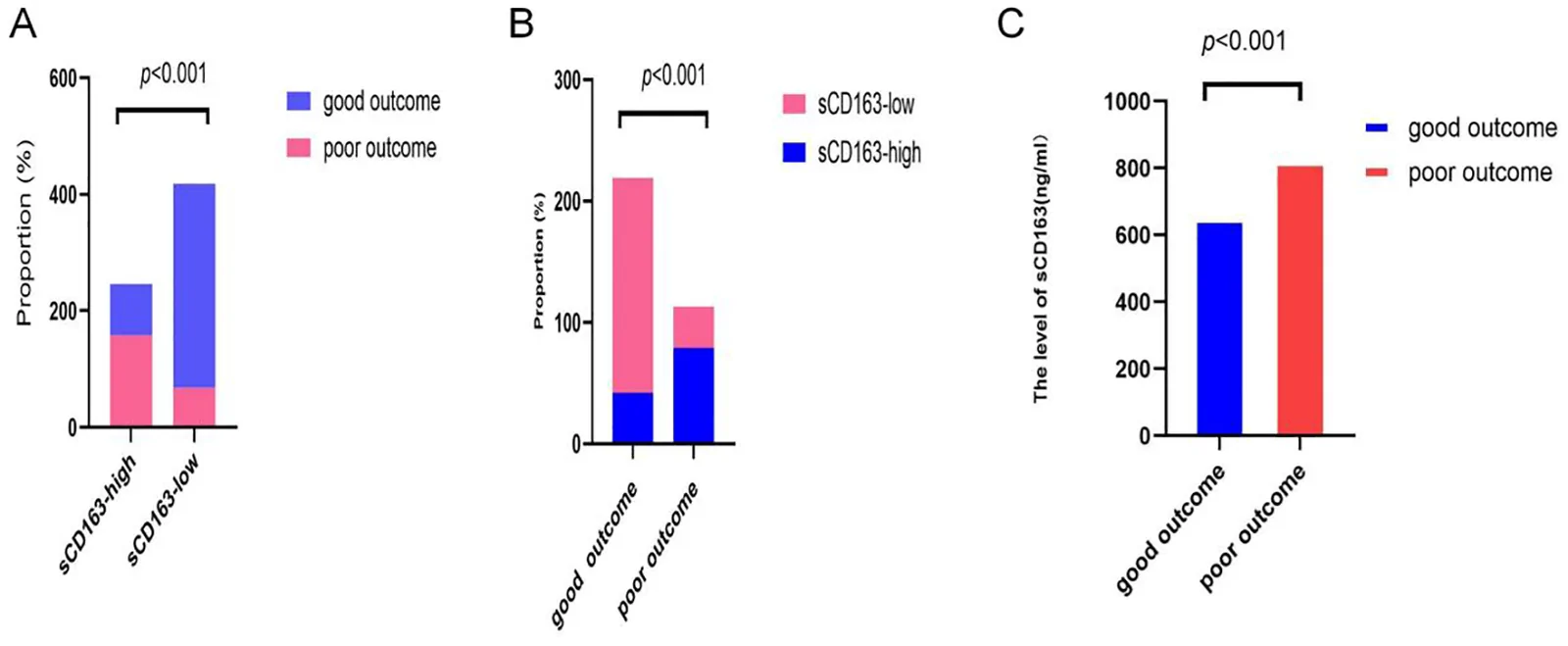
Serum sCD163 level is significantly elevated in patients with poor functional outcome at 3 months. Comparison of outcome proportions between sCD163 subgroups **(A)**. Comparison of high and low sCD163 proportions between good outcome and poor outcome groups **(B)**. Comparison of serum sCD163 levels between patients with good outcome and those with poor outcome **(C)**.

Serum sCD163 was markedly higher in the poor-outcome group than in the good-outcome group (820 ± 110 ng/mL vs. 650 ± 80 ng/mL, *t* = 20.59, *P* < 0.001) (**Figure 2C**). Using the optimal cutoff value of 742.5 ng/mL derived from ROC analysis, high sCD163 (≥ 742.5 ng/mL) was detected in 20.1% (88/438) of patients with good recovery and 69.9% (158/226) of poor-outcome patients (χ²= 156.3, *P* < 0.001) (**Figure 2B**). Among individuals with low sCD163, the incidence of poor prognosis was only 16.3% (68/418), while poor outcome occurred in 64.2% (158/246) of patients with elevated sCD163 (χ²= 147.2, *P* < 0.001) (**Figure 2A**).

### ROC analysis and optimal cutoff of serum sCD163

**Figure 3:**
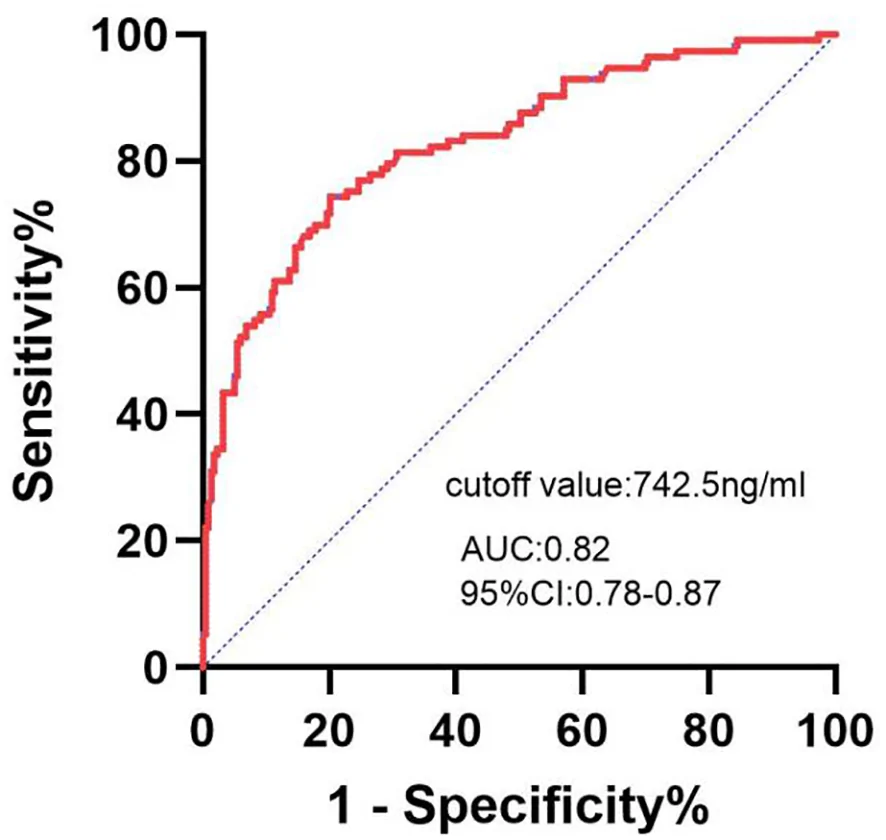
ROC curve of serum sCD163 for predicting 3-month poor functional outcome. The area under the curve (AUC) was 0.82 (95% CI: 0.78–0.87). The optimal cutoff value was 742.5 ng/mL, with a sensitivity of 69.9% and specificity of 79.9%.

ROC curve analysis in the training cohort revealed that serum sCD163 effectively predicted 3-month unfavorable outcome with an area under the curve (AUC) of 0.82 (95% CI: 0.78–0.87). The optimal cutoff value was 742.5 ng/mL, with a corresponding sensitivity of 69.9% and specificity of 79.9% (**Figure 3**). Based on this threshold, all participants were split into high- and low-sCD163 subgroups for subsequent regression analysis.

### Predictor selection

Candidate predictors were pre-specified based on clinical relevance and prior literature. The pre-specified candidate pool included age, sex, hypertension, diabetes, atrial fibrillation, baseline NIHSS score, DWI infarct volume, random blood glucose, white blood cell count, neutrophil percentage, platelet count, fibrinogen, international normalized ratio (INR), onset-to-admission time, TOAST subtypes, and serum sCD163. Variable selection was performed using least absolute shrinkage and selection operator (LASSO) regression with 10-fold cross-validation, employing the “1-standard error” (1-SE) rule to select the optimal penalty parameter λ. The LASSO procedure identified six predictors with non-zero coefficients: serum sCD163, baseline NIHSS score, DWI infarct volume, atrial fibrillation, age, and TOAST subtypes. These six variables were subsequently entered into a multivariable logistic regression model. After multivariable adjustment, four variables remained as independent predictors of 3-month poor functional outcome: serum sCD163, baseline NIHSS score, DWI infarct volume, and atrial fibrillation; age and TOAST subtypes lost statistical significance and were not retained in the final model (**Table 2**). Collinearity diagnostics revealed no significant multicollinearity among any candidate predictors, with variance inflation factors (VIF) ranging from 1.08 to 1.42 (all < 5), indicating no significant multicollinearity (**Supplementary Table 8**). Interaction tests revealed no substantial interactions among the independent variables (all *P* for interaction > 0.1) (**Supplementary Table 9**).

**Table 2:** Univariable and multivariable logistic regression analysis for predicting 3-month poor outcome (training set, n=664).

Variable	Univariable analysis		Multivariable analysis	
	OR (95% CI)	*P* value	OR (95% CI)	*P* value
sCD163 (high vs low)	9.24 (6.40–13.36)	<0.001	7.52 (4.42–12.78)	<0.001
Baseline NIHSS (per 1 point)	1.52 (1.38–1.68)	<0.001	1.35 (1.20–1.52)	<0.001
DWI infarct volume (per 1 mL)	1.10 (1.07–1.13)	<0.001	1.06 (1.03–1.09)	<0.001
Age (per 1 year)	1.03 (1.01–1.05)	0.002	—	—
Atrial fibrillation (yes vs no)	1.44 (1.08–1.92)	0.014	1.58 (1.01–2.47)	0.045
TOAST subtype (ref: SVO)			—	—
– LAA	1.34 (0.88–2.04)	0.173	—	—
– CE	1.45 (0.96–2.19)	0.078	—	—
– Other	1.69 (0.84–3.40)	0.142	—	—
– Undetermined	1.40 (0.61–3.21)	0.430	—	—

### Univariate and multivariate logistic regression analysis

Univariate logistic regression identified high sCD163 as a powerful predictor of poor functional outcome (OR = 9.24, 95% CI: 6.40–13.36, *P* < 0.001). Other significant predictors included higher baseline NIHSS score (per 1 point: OR = 1.52, 95% CI: 1.38–1.68, *P* < 0.001), increased DWI infarct volume (per 1 mL: OR = 1.10, 95% CI: 1.07–1.13, *P* < 0.001), advanced age (per 1 year: OR = 1.03, 95% CI: 1.01–1.05, *P* = 0.002), and atrial fibrillation (OR = 1.44, 95% CI: 1.08–1.92, *P* = 0.014). Regarding TOAST subtypes, compared with small vessel occlusion (SVO) as the reference, neither LAA (OR = 1.34, 95% CI: 0.88–2.04, *P* = 0.173), cardioembolism (OR = 1.45, 95% CI: 0.96–2.19, *P* = 0.078), other determined etiology (OR = 1.69, 95% CI: 0.84–3.40, P = 0.142), nor undetermined etiology (OR = 1.40, 95% CI: 0.61–3.21, *P* = 0.430) reached statistical significance (**Table 2**).

**Figure 4:**
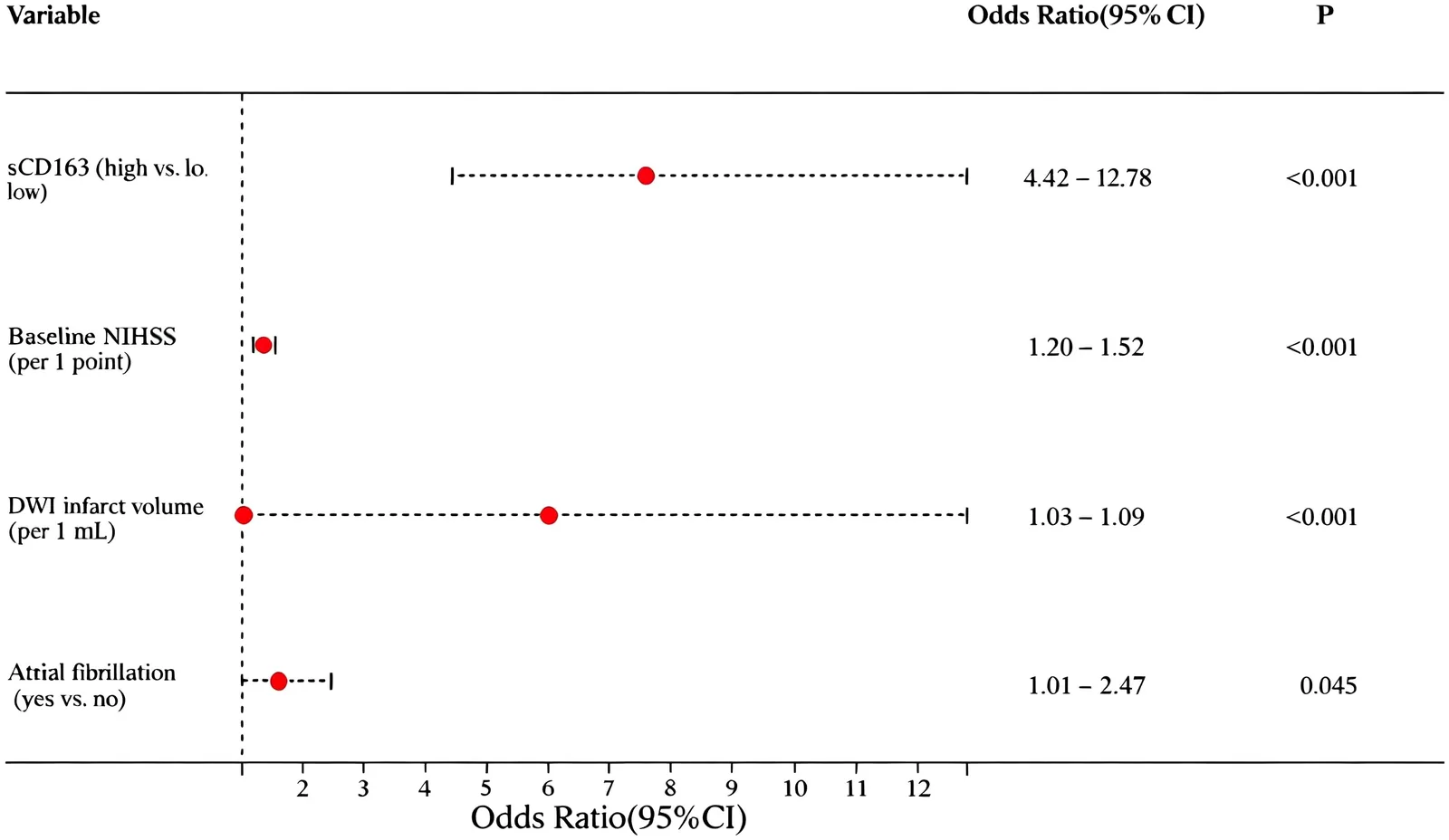
Forest plot of multivariable logistic regression analysis for predictors of 3-month poor functional outcome. The model was adjusted for age, baseline NIHSS score, DWI infarct volume, atrial fibrillation, and TOAST subtype.

After multivariable adjustment for potential confounders (age, baseline NIHSS, DWI volume, atrial fibrillation, and TOAST subtypes), elevated sCD163 (high vs. low) remained an independent risk marker for 3-month poor prognosis (adjusted OR = 7.52, 95% CI: 4.42–12.78, *P* < 0.001). In addition, baseline NIHSS (adjusted OR = 1.35, 95% CI: 1.20–1.52, *P* < 0.001), DWI infarct volume (adjusted OR = 1.06, 95% CI: 1.03–1.09, *P* < 0.001), and atrial fibrillation (adjusted OR = 1.58, 95% CI: 1.01–2.47, *P* = 0.045) were confirmed as independent predictors. Age (adjusted OR = 1.01, 95% CI: 0.99–1.04, *P* = 0.279) and all TOAST subtypes (LAA: adjusted OR = 1.21, 95% CI: 0.78–1.88, *P* = 0.395; CE: adjusted OR = 1.32, 95% CI: 0.86–2.03, *P* = 0.208; other determined: adjusted OR = 1.45, 95% CI: 0.70–3.00, *P* = 0.315; undetermined: adjusted OR = 1.28, 95% CI: 0.54–3.03, *P* = 0.576) lost statistical significance after adjustment (all *P* > 0.05) (**Table 2**; **Figure 4**).

The final predictive model for 3-month poor functional outcome (mRS > 2) was constructed as follows: y = 1/(1 + e^−z^), where y represents the predicted probability of poor functional outcome, e is the exponential function, and Z = −5.123 + 2.015·X_1_ + 0.298·X_2_ + 0.057·X_3_ + 0.455·X_4_. X_1_, X_2_, X_3_, and X_4_ represent serum sCD163 level (high vs. low), baseline NIHSS score (per 1 point), DWI infarct volume (per 1 mL), and history of atrial fibrillation (yes vs. no), respectively.

### Incremental predictive value of sCD163

**Figure 5:**
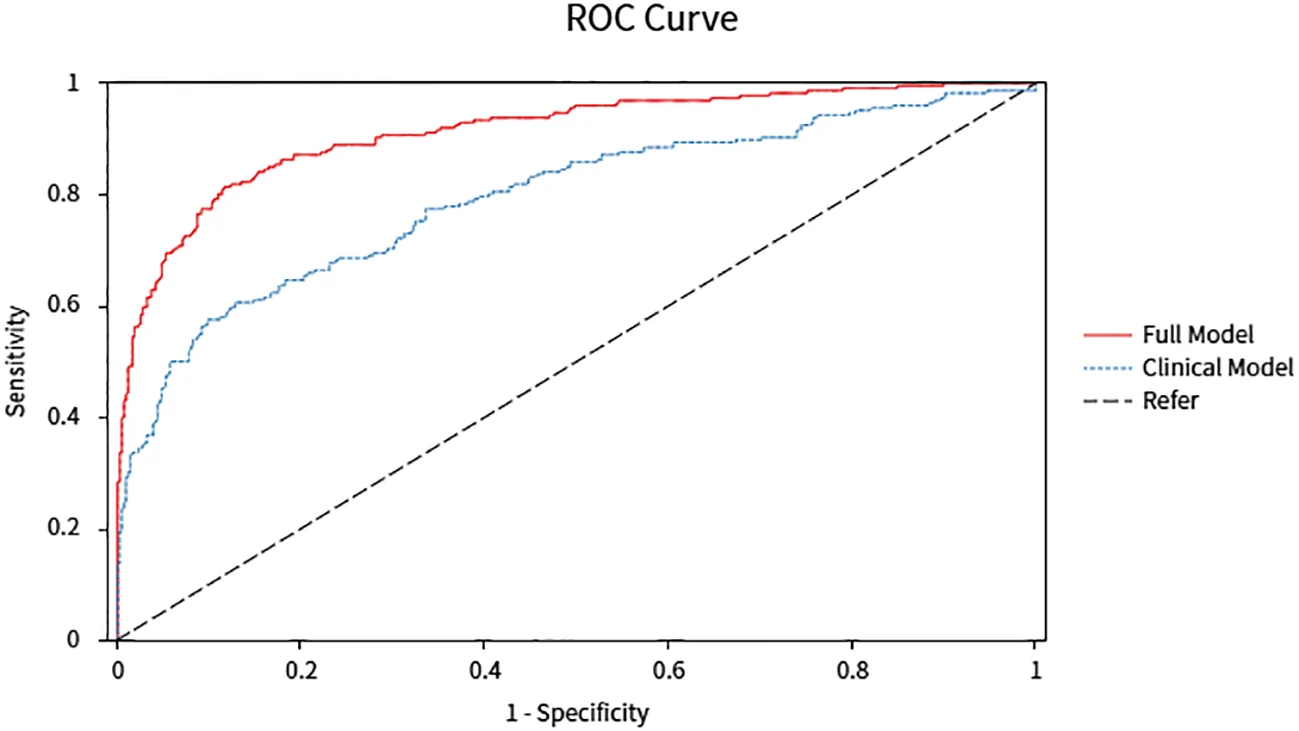
ROC curves of the clinical model and the full model for predicting 3-month poor functional outcome. The full model (sCD163 + NIHSS + DWI infarct volume + atrial fibrillation) showed superior discriminative performance (AUC = 0.913, 95% CI: 0.889–0.937) compared with the clinical model (AUC = 0.795, 95% CI: 0.757–0.833). DeLong test: ΔAUC = 0.118, 95% CI: 0.083–0.152, *P* < 0.001.

To evaluate the additional prognostic information provided by sCD163 beyond traditional predictors, we compared the clinical model (NIHSS + DWI volume + atrial fibrillation) with the full model (clinical + sCD163). The full model demonstrated significantly better discrimination than the clinical model, with an AUC of 0.913 (95% CI: 0.889–0.937) versus 0.795 (95% CI: 0.757–0.833) for the clinical model (ΔAUC = 0.118, DeLong test *P* < 0.001) (**Figure 5**). The likelihood ratio test also showed a significant improvement in model fit after adding sCD163 (χ² = 6.85, *P* = 0.009). In terms of risk reclassification, the addition of sCD163 yielded a significant net reclassification improvement (NRI = 0.218, 95% CI: 0.089–0.347, *P* = 0.003) and integrated discrimination improvement (IDI = 0.032, 95% CI: 0.014–0.050, *P* = 0.001), indicating that sCD163 correctly reclassified a substantial proportion of patients into appropriate risk categories (**Table 3**). All bootstrap-derived estimates were based on 1,000 resamples.

**Table 3:** Net reclassification and integrated discrimination improvement.

Index	Estimate	95% CI*	*P* value*
NRI (Clinical + sCD163 vs. Clinical model)	0.218	0.089 – 0.347	0.003
IDI	0.032	0.014 – 0.050	0.001

*Bootstrap-based estimates with 1,000 resamples. The clinical model included baseline NIHSS, DWI infarct volume, and atrial fibrillation. Abbreviations: CI, confidence interval; IDI, integrated discrimination improvement; NRI, net reclassification improvement.

### Internal validation of the predictive model

**Figure 6:**
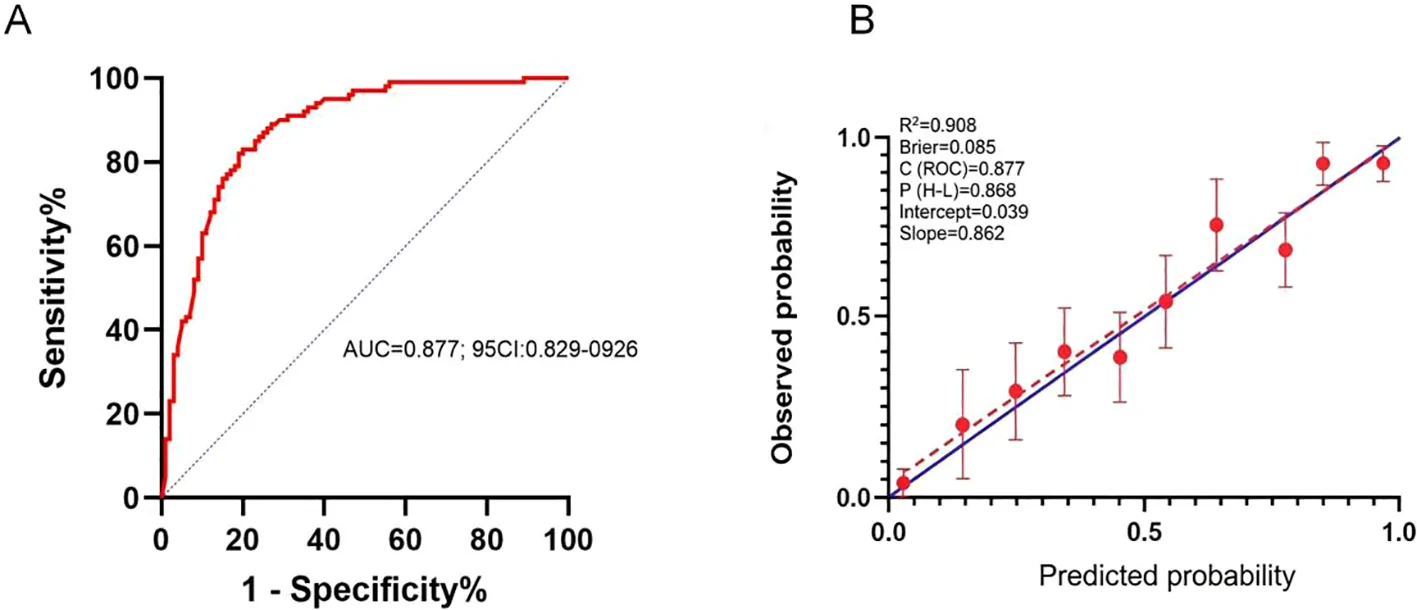
Internal validation of the prediction model via bootstrap resampling (1,000 iterations). **(A)** Bootstrap-corrected receiver operating characteristic (ROC) curve of the model; the AUC was 0.877 (95% CI: 0.829 -0.926). **(B)** Calibration plot demonstrating the consistency between predicted probabilities and observed probabilities. The Hosmer-Lemeshow test showed *P* = 0.868, indicating satisfactory calibration of the model.

Bootstrap internal validation (1000 resampling iterations) was performed to evaluate model performance. The optimism-corrected AUC was 0.877 (95% CI: 0.829–0.926), with R² = 0.908 and Brier score = 0.085. Calibration analysis showed a calibration intercept of 0.039 (95% CI: -0.127 to 0.205) and a calibration slope of 0.862 (95% CI: 0.781–0.943), both close to ideal values (intercept = 0, slope = 1); the Hosmer-Lemeshow test yielded *P* = 0.868 (**Figure 6**). Collectively, the established combined model exhibited favorable discrimination and good calibration without obvious overfitting.

### External validation of the predictive model

**Figure 7:**
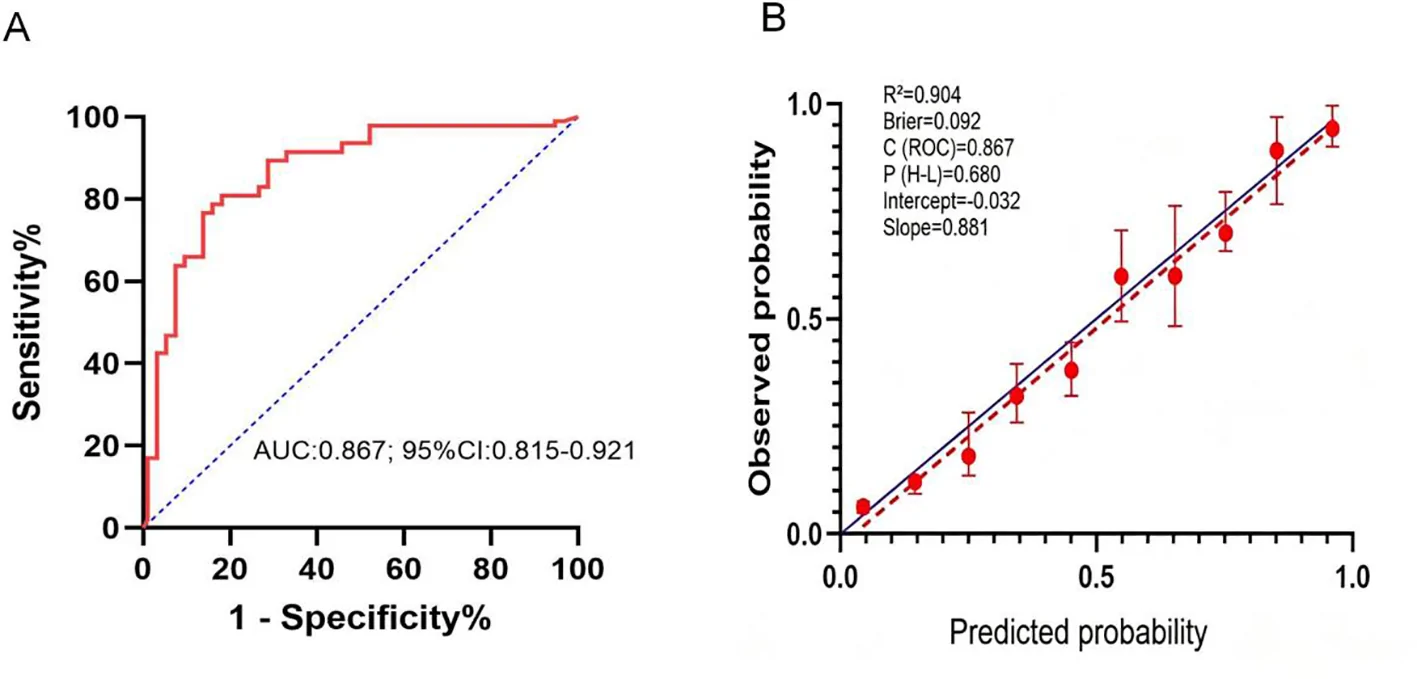
External validation of the predictive model using the independent validation cohort (n = 300). **(A)** Receiver operating characteristic (ROC) curve of the predictive model in the validation cohort. The AUC was 0.867 (95% CI: 0.815–0.921). **(B)** Calibration plot showing the agreement between predicted probabilities and observed frequencies. The Hosmer-Lemeshow test yielded *P* = 0.680.

External validation was performed by directly applying the original model coefficients to the independent validation cohort (n = 300) without recalibration, as recommended by the TRIPOD guidelines. The optimism-corrected AUC was 0.867 (95% CI: 0.815–0.921), with R² = 0.904 and Brier score = 0.092. Calibration analysis showed a calibration intercept of 0.032 (95% CI: −0.125 to 0.189) and a calibration slope of 0.881 (95% CI: 0.805–0.957), both close to ideal values (intercept = 0, slope = 1); the Hosmer-Lemeshow test yielded *P* = 0.680 (**Figure 7**). Collectively, the established combined model maintained favorable discrimination and good calibration in the independent validation cohort.

### Decision curve analysis

**Figure 8:**
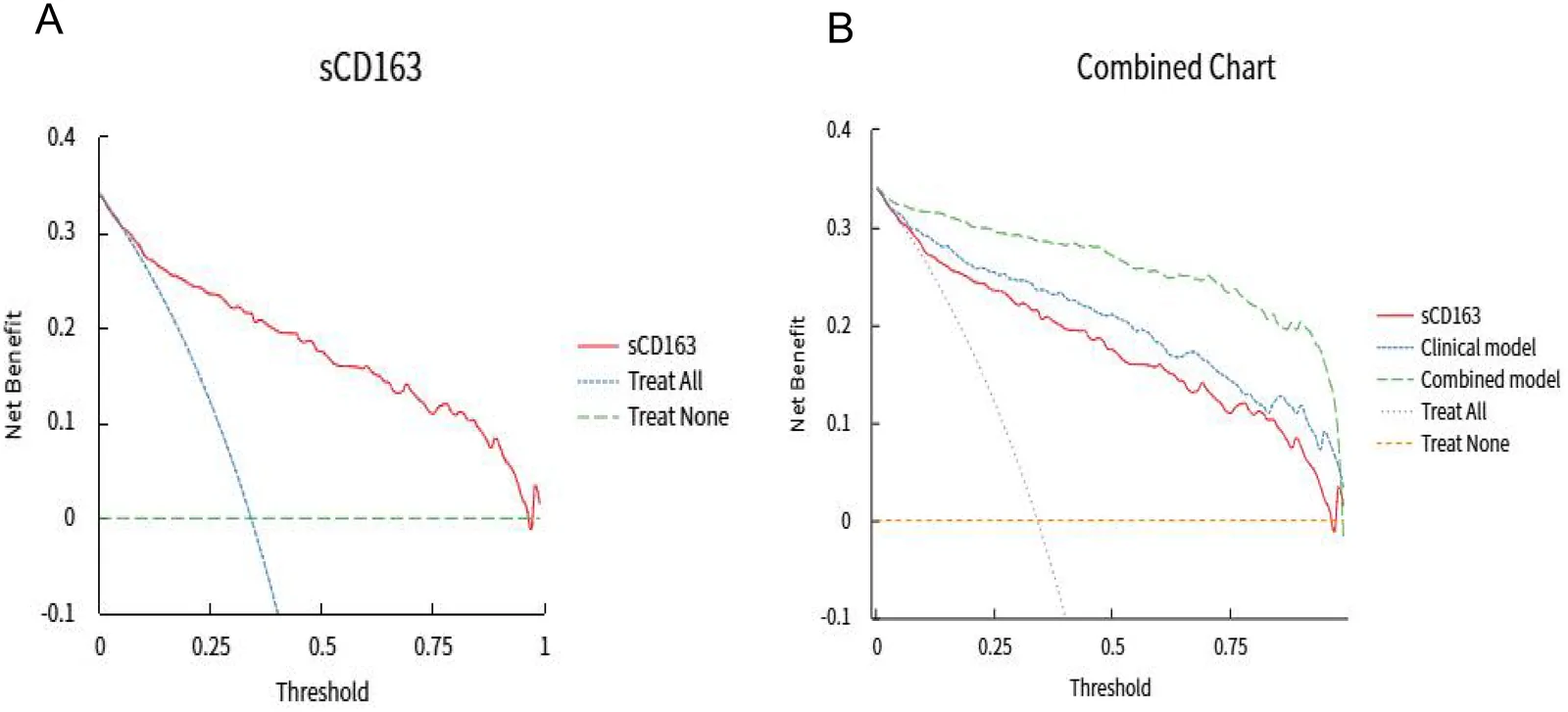
Decision curve analysis for predicting 3-month poor functional outcome. Decision curves of the univariate prediction model based on serum sCD163 level **(A)** and of sCD163, the clinical model, and the full model incorporating sCD163 **(B)** are shown. The analysis demonstrates that the full model provides greater clinical net benefit across a range of threshold probabilities.

Decision curve analysis (DCA) demonstrated that the full predictive model incorporating sCD163 yielded superior net clinical benefit across most threshold probability ranges compared with the clinical-only model and the two extreme intervention strategies (treat-all or treat-none), indicating favorable clinical application value of the biomarker (**Figure 8**).

### Sensitivity analyses

To assess the robustness of our findings, we performed two sensitivity analyses. First, to address potential survivor bias, we repeated the analysis by including the 10 patients who died within 48 hours of admission. For these patients, the 3-month mRS was assigned a value of 6 (death), and missing sCD163 values were handled using multiple imputation. The re-analysis with the expanded sample (n = 674) yielded results consistent with the primary analysis: high sCD163 remained an independent predictor of poor outcome (aOR = 6.98, 95% CI: 4.08–11.93, *P* < 0.001), with a bootstrap-corrected AUC of 0.862 (95% CI: 0.815–0.909). These findings further confirm that the exclusion of early death patients did not materially bias our results (**Supplementary Table 3**).

Second, to address the potential overoptimism associated with ROC-derived dichotomization, we treated sCD163 as a continuous variable (per 10 ng/mL increase). After adjusting for the same covariates, sCD163 remained an independent predictor of 3-month poor functional outcome (aOR = 1.08, 95% CI: 1.05–1.11, *P* < 0.001). The bootstrap-corrected AUC for the continuous model was 0.848 (95% CI: 0.798–0.898), which was slightly lower than but comparable to the primary dichotomized model (bootstrap-corrected AUC = 0.877, 95% CI: 0.829–0.926) (**Supplementary Table 4**). The modest difference (ΔAUC = 0.029) between the two coding strategies suggests that the ROC-derived cutoff did not substantially overestimate the predictive performance of sCD163, confirming the robustness of our findings regardless of sCD163 coding format.

## Discussion

Using a prospective design with a training cohort and an independent external validation cohort, this study systematically evaluated the predictive value of serum sCD163 measured within 24 hours of onset for poor functional outcome at 3 months in AIS patients. Our results showed that serum sCD163 level was significantly positively associated with poor 3-month outcome, and a high sCD163 level was an independent risk marker for poor outcome. Moreover, the prediction model combining sCD163, baseline NIHSS score, and DWI infarct volume yielded an internal bootstrap-validated AUC of 0.877 (95% CI: 0.829–0.926) and an independent external validation AUC of 0.867 (95% CI: 0.815–0.921), together with good calibration and clinical net benefit, providing a reliable tool for early risk stratification of AIS patients.

One core finding of this study was that serum sCD163 measured within 24 hours of admission was independently associated with poor functional outcome at 3 months. Multivariable logistic regression showed that patients with a high sCD163 level had a 7.52-fold increased risk of poor outcome compared with those with a low level (adjusted OR = 7.52, 95% CI: 4.42 –12.78, *P* < 0.001). While this strong association is noteworthy, it is important to recognize that circulating sCD163 primarily reflects systemic macrophage activation; whether this peripheral signal directly represents neuroinflammation in the ischemic brain, or whether it reflects a systemic inflammatory state that secondarily influences stroke outcomes, remains to be elucidated. This result is consistent with previous clinical studies. Sun et al. prospectively enrolled 300 AIS patients and found that plasma sCD163 concentration was positively correlated with improvement in NIHSS score and negatively correlated with the risk of poor functional outcome during follow-up, suggesting that sCD163 may serve as a potential biomarker associated with disease severity and functional outcome (16). Notably, Kang et al. analyzed thrombus pathology in 89 AIS patients with large vessel occlusion who underwent endovascular treatment and found that both CD163 and von Willebrand factor were significantly associated with 90-day poor outcome in patients treated with remedial stenting; the AUC of CD163 for predicting poor outcome was 0.7639, and the adjusted OR was 4,838.64 (95% CI: 1.86–1.26 × 10^7^) (7). These studies provide evidence from both peripheral blood and thrombus pathology confirming the value of CD163/sCD163 in AIS prognosis. Furthermore, Bhattacharya et al. systematically reviewed the potential of sCD163 as a prognostic biomarker and therapeutic target in stroke, emphasizing its role in modulating hemoglobin and heme bioavailability (12). Sahebi et al. further highlighted the dual role of CD163 in post-stroke efferocytosis (clearance of apoptotic cells) and tissue repair, noting that persistent overactivation of macrophages could potentially aggravate secondary injury through the release of reactive oxygen species and matrix metalloproteinases (20). On the basis of a large sample size and a dual-cohort design with strict exclusion of reperfusion therapy, our study further systematically validates the independent prognostic association of sCD163 with long-term functional outcome in AIS, providing high-quality evidence for clinical translation of this biomarker.

The pathophysiological mechanisms through which sCD163 may be involved in the regulation of AIS prognosis are complex and not fully established; however, several lines of evidence suggest the following three core aspects. First, CD163 is a specific marker of monocyte/macrophage activation. After AIS onset, cerebral ischemia-induced tissue injury can rapidly activate peripheral blood monocytes and brain microglia, and large numbers of CD163^+^ macrophages have been observed to infiltrate the ischemic brain tissue, where they may participate in inflammatory responses and tissue repair (11, 13). Using RNA-sequencing analysis, Rajan et al. found that CD163^+^ cells isolated from ischemic brain tissue were enriched for cell-cycle-related functional groups at day 3 after ischemia; flow cytometry confirmed an increase in CD163^+^ cell numbers, and Ki67-positive CD163^+^ cells were increased in the perivascular space (11). CD163^+^ cells up-regulated multiple inflammatory genes in the ischemic parenchyma and expressed inducible nitric oxide synthase (iNOS), suggesting that they may acquire a pro-inflammatory phenotype after stroke. Shedding of CD163 from the macrophage surface releases sCD163 into the peripheral blood, and serum sCD163 levels may directly reflect the degree of macrophage activation and the intensity of inflammation (9, 10). Second, excessive post-stroke inflammation is considered a core mechanism potentially leading to secondary brain injury and aggravated neurological deficits. CD163^+^ central nervous system border-associated macrophages undergo reprogramming of their gene expression profile after acute ischemic stroke, rapidly affecting leukocyte chemotaxis and blood-brain barrier integrity and modulating neurological damage during the acute phase (14, 15). Inflammatory cytokine storms have been reported to disrupt the blood-brain barrier, induce neuronal apoptosis, and exacerbate brain edema, ultimately potentially affecting long-term functional recovery (11, 13). As a stable biomarker of inflammation, elevated sCD163 levels may indicate excessive activation of the inflammatory response and could be associated with an increased risk of secondary brain injury and poor outcome. Third, CD163 is involved in hemoglobin clearance and cerebral iron metabolism. After AIS, extravasation of red blood cells releases large amounts of hemoglobin, and CD163, as a scavenger receptor, can efficiently clear the hemoglobin-haptoglobin complex, potentially reducing iron overload-induced oxidative stress and neuronal injury. Bhattacharya et al. noted that the haptoglobin-CD163-heme oxygenase-1 (HO-1) pathway may play a key role in promoting clearance of hemoglobin and its toxic products (12); the review by Sahebi et al. also systematically summarized the central role of CD163 in post-stroke iron metabolism and oxidative stress regulation (20). Together, these mechanisms provide a plausible biological rationale for sCD163 as a prognostic biomarker in AIS, although direct evidence linking circulating sCD163 to these specific CNS pathways remains limited and requires further investigation.

Another important finding of this study was that the combined model incorporating sCD163, baseline NIHSS score, DWI infarct volume, and atrial fibrillation achieved an internal bootstrap-validated AUC of 0.877 (95% CI: 0.829–0.926) and an independent external validation AUC of 0.867 (95% CI: 0.815–0.921). The Hosmer-Lemeshow test P-values were 0.868 in the training cohort and 0.680 in the validation cohort, indicating good calibration. These results suggest that adding the specific macrophage activation marker sCD163 to traditional clinical and imaging indices may effectively improve the accuracy of prognostic prediction in AIS.

Currently, the discriminative performance (AUC) of AIS prognostic models based solely on traditional indices or composite inflammatory markers is mostly in the range of 0.7–0.8. For example, Li et al. retrospectively analyzed 762 AIS patients receiving intravenous thrombolysis and found that the AUCs of the neutrophil-to-lymphocyte ratio (NLR) and the systemic immune-inflammation index (SII) for predicting poor 3-month outcome were 0.707 and 0.715, respectively (17). A meta-analysis of the lymphocyte-to-monocyte ratio (LMR) also confirmed the significant association of peripheral blood inflammatory indices with AIS prognosis, but the overall predictive performance remained limited (8, 18). In contrast, our model, which includes sCD163 – a marker that directly reflects the specific activation state of macrophages – achieved an AUC of approximately 0.87, significantly outperforming the composite inflammatory indices. This observation suggests that macrophage-specific markers may provide unique incremental value in prognostic assessment.

Notably, Yang et al. constructed a nomogram for predicting 3-month outcome in elderly patients with non-valvular atrial fibrillation-induced AIS and reported an AUC of 0.933 (21). That model had higher predictive performance than ours, but its applicability is restricted to patients with cardioembolic stroke due to atrial fibrillation. Our model, in contrast, is applicable to AIS patients with all etiologies (including LAA, CE, and SVO subtypes) who have not received reperfusion therapy, and it has undergone independent external validation, offering broader generalizability. Liu et al. externally validated the iScore, ASTRAL, DRAGON, and THRIVE scores in 439 patients with large-vessel occlusion AIS, reporting AUCs of 0.8475, 0.8324, 0.8196, and 0.8039, respectively (6). These AUCs are similar to that of our model, but our model additionally provides an inflammation-related biological dimension (sCD163) that may be targetable for future therapeutic interventions. In summary, our model maintains good discriminative ability while offering etiologic generalizability and mechanistic interpretability.

This study has several limitations. First, although the external validation cohort was collected from an independent center, both the training and validation centers are located in the same city and are tertiary teaching hospitals. Therefore, the generalizability of our model to different geographic regions, non-teaching or community hospitals, and more diverse ethnic populations still requires confirmation through multi-center external validation involving a broader range of settings. Second, we measured sCD163 only once (within 24 hours of admission) and did not capture its dynamic changes over time. Future studies could employ multi-point measurements (e.g., admission, day 3, day 7) to explore the relationship between sCD163 trajectories and long-term functional recovery.

Third, we did not perform mechanistic experiments to clarify the specific pathophysiological role of CD163/sCD163 in AIS development. Future studies using cellular and animal models are needed to elucidate the regulatory mechanisms of sCD163 in post-stroke neuroinflammation and secondary brain injury, and to determine whether the observed association between circulating sCD163 and poor outcome is causal or reflects an epiphenomenon of systemic inflammation. Fourth, our findings are primarily applicable to a selected subgroup of patients with acute ischemic stroke. Patients who received reperfusion therapy were excluded to reduce confounding effects on inflammatory markers and functional outcomes; those who died within 48 hours of admission were excluded from the primary analysis (although sensitivity analysis confirmed that this exclusion did not materially bias our results); and patients with mild (NIHSS < 4) or severe (NIHSS > 22) strokes were not included to ensure a more homogeneous study population. In addition, patients with active infection, autoimmune diseases, severe organ dysfunction, or malignancy were excluded because these conditions may confound sCD163 levels. Therefore, our results should not be automatically generalized to all patients with AIS, and future studies are needed to validate our findings in broader patient populations, including those receiving reperfusion therapy, those with mild or severe strokes, and those in different healthcare settings or geographic regions. Fifth, some potential confounders, such as collateral circulation status, hemorrhagic transformation, white matter hyperintensity burden, early neurological deterioration, rehabilitation intensity, and additional inflammatory markers (e.g., CRP, IL-6), were not included in the analysis due to data unavailability. Incorporating these variables in future studies could further improve predictive performance and provide a more comprehensive prognostic model for AIS patients.

In conclusion, through a large-scale prospective dual-cohort study with independent external validation, this study demonstrates a strong association between serum sCD163 level within 24 hours of onset and poor functional outcome at 3 months in AIS patients. The multivariable logistic regression model combining sCD163, baseline NIHSS score, DWI infarct volume, and atrial fibrillation (internal validation AUC = 0.877, external validation AUC = 0.867) can accurately identify AIS patients at risk of poor prognosis and has important clinical application value. While our findings support sCD163 as a promising prognostic biomarker, the underlying mechanisms remain to be fully elucidated. Future multi-center prospective studies are needed to further validate our findings, to explore the pathophysiological mechanisms of sCD163 in AIS, and to investigate its prognostic value in reperfusion-treated populations, as well as to clarify whether the observed associations reflect causal pathways or systemic inflammatory epiphenomena, thereby providing new targets and clinical tools for individualized treatment and prognostic assessment of AIS patients.

## Data Availability

The original contributions presented in the study are included in the article/**Supplementary Material**. Further inquiries can be directed to the corresponding author.
