# Supplementary material for: Serum sCD163 as a potential biomarker for predicting poor functional outcome at 3 months in acute ischemic stroke without reperfusion: a prospective study
Source: Front Immunol. 2026 Aug 11;17:1914293. doi: 10.3389/fimmu.2026.1914293 (PMC13503311; doi:10.3389/fimmu.2026.1914293)
Supplement: Supplementary file 1 [file DataSheet1.pdf]

Supplementary Material

Supplementary Table S1 Baseline characteristics of the training cohorts

Variable	Good outcome (mRS≤2, n=438)	Poor outcome (mRS>2, n=226)	Statistic ($t/\chi^2/Z$)	P value
Age (years, mean±SD)	65.2 ± 11.3	66.5 ± 10.8	t = 1.45	0.148
Male, n (%)	285 (65.1)	145 (64.2)	χ^2 = 0.058	0.810
Smoking, n (%)	166 (37.9)	82 (36.3)	χ^2 = 0.17	0.680
BMI [M (Q1, Q3)]	24.2 (22.5, 26.3)	24.4 (22.8, 26.6)	Z = 0.81	0.428
Alcohol consumption, n (%)	106 (24.2)	58 (25.7)	χ^2 = 0.18	0.672
Hypertension, n (%)	267 (61.0)	149 (65.9)	χ^2 = 1.98	0.158
Hypercholesterolemia, n (%)	180 (41.1)	96 (42.5)	χ^2 = 0.123	0.726
Diabetes, n (%)	124 (28.3)	62 (27.4)	χ^2 = 0.06	0.808
Atrial fibrillation, n (%)	96 (21.9)	71 (31.4)	χ^2 = 6.07	0.014
Family history of stroke, n (%)	59 (13.5)	32 (14.2)	χ^2 = 0.06	0.807

Variable	Good outcome (mRS≤2, n=438)	Poor outcome (mRS>2, n=226)	Statistic ($t/\chi^2/Z$)	P value
Antiplatelet drug use, n (%)	199 (45.4)	99 (43.8)	$\chi^2 = 0.16$	0.691
Anticoagulant drug use, n (%)	68 (15.5)	37 (16.4)	$\chi^2 = 0.09$	0.766
Baseline NIHSS [M (Q1, Q3)]	7 (5, 10)	12 (9, 17)	Z = 2.66	0.007
DWI hyperintensity volume (ml, mean±SD)	15.2 ± 8.5	28.5 ± 14.0	t = 13.09	<0.001
Random blood glucose (mmol/L, mean±SD)	7.98 ± 2.78	8.12 ± 2.90	t = 0.60	0.547
White blood cell count ($\times 10^9$/L, mean±SD)	8.35 ± 2.95	8.50 ± 3.10	t = 0.60	0.548
Neutrophil percentage (%, mean±SD)	0.710 ± 0.11	0.716 ± 0.12	t = 0.64	0.522
Platelet count ($\times 10^9$/L, mean±SD)	217 ± 55	220 ± 58	t = 0.67	0.504
Fibrinogen (g/L, mean±SD)	3.25 ± 0.78	3.28 ± 0.81	t = 0.47	0.637
INR (mean±SD)	1.03 ± 0.07	1.04 ± 0.08	t = 1.67	0.095
Infarct location, n (%)			$\chi^2 = 0.89$	0.346
– Anterior circulation	250 (57.1)	129 (57.1)		

Variable	Good outcome (mRS≤2, n=438)	Poor outcome (mRS>2, n=226)	Statistic ($t/\chi^2/Z$)	P value
– Posterior circulation	188 (42.9)	97 (42.9)		
Distribution of culprit vessels, n (%)			χ^2 = 0.77	0.681
– Internal carotid artery	37 (8.4)	19 (8.4)		
– Middle cerebral artery	227 (51.8)	117 (51.8)		
– Basilar artery	174 (39.8)	90 (39.8)		
Vessel stenosis degree, n (%)			χ^2 = 0.96	0.619
– Normal	64 (14.6)	32 (14.2)		
– Stenosis	141 (32.2)	73 (32.3)		
– Occlusion	233 (53.2)	121 (53.5)		
Onset-to-admission time (h) [M (Q1, Q3)]	8.0 (4.5, 13.5)	8.5 (5.0, 14.0)	Z = 0.67	0.504
TOAST classification, n (%)			χ^2 = 4.38	0.357
– Large artery atherosclerosis	162 (37.0)	94 (41.6)		
– Cardioembolism	118 (26.9)	64 (28.3)		

Variable	Good outcome (mRS≤2, n=438)	Poor outcome (mRS>2, n=226)	Statistic ($t/\chi^2/Z$)	P value
– Small vessel occlusion	106 (24.2)	42 (18.6)		
– Other determined etiology	33 (7.5)	15 (6.6)		
– Undetermined etiology	19 (4.3)	11 (4.9)		
sCD163 (ng/mL, mean±SD)	650 ± 80	820 ± 110	t = 20.59	<0.001

Supplementary Table S2. Baseline characteristics of the validation cohort

Variable	Good outcome (mRS≤2, n=198)	Poor outcome (mRS>2, n=102)	Statistic ($t/\chi^2/Z$)	P value
Age (years, mean±SD)	64.8 ± 11.6	66.3 ± 11.2	t = 1.07	0.285
Male, n (%)	126 (63.6)	66 (64.7)	χ^2 = 0.034	0.853
Smoking, n (%)	75 (37.9)	36 (35.3)	χ^2 = 0.19	0.663
BMI [M (Q1, Q3)]	24.1 (22.4, 26.2)	24.4 (22.7, 26.5)	Z = 0.67	0.503

Alcohol consumption, n (%)	51 (25.8)	24 (23.5)	χ^2 = 0.18	0.672
Hypertension, n (%)	123 (62.1)	65 (63.7)	χ^2 = 0.074	0.785
Hypercholesterolemia, n (%)	84 (42.4)	40 (39.2)	χ^2 = 0.28	0.597
Diabetes, n (%)	53 (26.8)	29 (28.4)	χ^2 = 0.09	0.765
Atrial fibrillation, n (%)	38 (19.2)	33 (32.4)	χ^2 = 6.12	0.013
Family history of stroke, n (%)	29 (14.6)	13 (12.7)	χ^2 = 0.21	0.647
Antiplatelet drug use, n (%)	91 (46.0)	43 (42.2)	χ^2 = 0.39	0.532
Anticoagulant drug use, n (%)	30 (15.2)	17 (16.7)	χ^2 = 0.11	0.740
Baseline NIHSS [M (Q1, Q3)]	7 (5, 10)	12 (8, 16)	Z = 2.51	0.012
DWI hyperintensity volume (ml, mean±SD)	15.0 ± 8.8	27.0 ± 14.2	t = 8.11	<0.001
Random blood glucose (mmol/L, mean±SD)	8.28 ± 2.95	8.36 ± 3.02	t = 0.22	0.824
White blood cell count ($\times 10^9$/L, mean±SD)	8.60 ± 3.15	8.66 ± 3.22	t = 0.16	0.873
Neutrophil percentage (%, mean±SD)	0.726 ± 0.12	0.732 ± 0.13	t = 0.40	0.691

Platelet count (×10⁹/L, mean±SD)	220 ± 58	223 ± 61	t = 0.42	0.674
Fibrinogen (g/L, mean±SD)	3.22 ± 0.76	3.25 ± 0.78	t = 0.32	0.747
INR (mean±SD)	1.04 ± 0.08	1.05 ± 0.09	t = 0.97	0.334
Infarct location, n (%)			χ^2 = 0.52	0.471
– Anterior circulation	122 (61.6)	60 (58.8)		
– Posterior circulation	76 (38.4)	42 (41.2)		
Distribution of culprit vessels, n (%)			χ^2 = 0.61	0.737
– Internal carotid artery	14 (7.1)	7 (6.9)		
– Middle cerebral artery	108 (54.5)	54 (52.9)		
– Basilar artery	76 (38.4)	41 (40.2)		
Vessel stenosis degree, n (%)			χ^2 = 0.82	0.665
– Normal	26 (13.1)	13 (12.7)		
– Stenosis	62 (31.3)	32 (31.4)		
– Occlusion	110 (55.6)	57 (55.9)		
Onset-to-admission time (h) [M	8.0 (4.5, 13.5)	8.0 (4.5, 14.0)	Z = 0.18	0.857

(Q1, Q3)]				
**TOAST classification, n (%)**			**χ^2 = 4.18**	**0.382**
– Large artery atherosclerosis	76 (38.4)	48 (47.1)		
– Cardioembolism	58 (29.3)	29 (28.4)		
– Small vessel occlusion	45 (22.7)	17 (16.7)		
– Other determined etiology	10 (5.1)	4 (3.9)		
– Undetermined etiology	9 (4.5)	4 (3.9)		
sCD163 (ng/mL, mean±SD)	658 ± 78	797 ± 115	t = 10.98	<0.001

Supplementary Table S3. Sensitivity analysis for the inclusion of early death patients

	n	sCD163 aOR (95% CI)	P value*	AUC (95% CI)*	ΔAUC vs. primary analysis
Primary analysis (excluding early death)	664	7.52 (4.42–12.78)	<0.001	0.877 (0.829–0.926)	—
Sensitivity analysis (including early death)*	674	6.98 (4.08–11.93)	<0.001	0.862 (0.815–0.909)	−0.015

*For patients who died within 48 hours of admission (n = 10), 3-month mRS was assigned a value of 6 (death), and missing sCD163 values were handled using multiple imputation. The model was adjusted for the same covariates as the primary analysis (baseline NIHSS, DWI infarct volume, and atrial fibrillation). All AUCs were optimism-corrected using bootstrap resampling with 1,000 iterations to ensure comparability with the primary analysis.

Abbreviations: aOR, adjusted odds ratio; AUC, area under the receiver operating characteristic curve; CI, confidence interval; DWI, diffusion-weighted imaging; mRS, modified Rankin Scale; NIHSS, National Institutes of Health Stroke Scale; sCD163, soluble CD163.

Supplementary Table S4. Sensitivity analysis: sCD163 as a continuous variable

Variable	aOR	95% CI	P* value
sCD163 (per 10 ng/mL increase)	1.08	1.05–1.11	< 0.001
Baseline NIHSS (per 1 point)	1.34	1.19–1.51	< 0.001
DWI infarct volume (per 1 mL)	1.07	1.04–1.10	< 0.001
Atrial fibrillation (yes vs. no)	1.59	1.02–2.49	0.040
Age (per 1 year)	1.02	0.99–1.04	0.201
**TOAST subtype (ref: SVO)**			
– Large artery atherosclerosis (LAA)	1.20	0.75–1.92	0.450

Variable	aOR	95% CI	P* value
– Cardioembolism (CE)	1.62	0.98–2.68	0.061
– Other determined etiology	1.33	0.63–2.81	0.458
**Model performance**			
Optimism-corrected AUC (95% CI)*	**0.848**	0.798–0.898	

*Bootstrap-corrected AUC with 1,000 resamples, adjusted for the same covariates as the primary analysis (baseline NIHSS, DWI infarct volume, and atrial fibrillation).

Supplementary Table S5. LASSO regression coefficient paths and variable selection

Variable	Category	Coefficient at optimal λ	Selected by LASSO
**Demographics**			
Age	Demographics	0.018	✓
Sex	Demographics	0.000	✕
**Vascular risk factors**			

Variable	Category	Coefficient at optimal λ	Selected by LASSO
Hypertension	Comorbidity	0.000	✕
Diabetes	Comorbidity	0.000	✕
Atrial fibrillation	Comorbidity	0.312	✓
**Clinical severity and imaging**			
Baseline NIHSS	Clinical	0.168	✓
DWI infarct volume	Imaging	0.089	✓
**Laboratory tests**			
Random blood glucose	Laboratory	0.000	✕
White blood cell count	Laboratory	0.000	✕
Neutrophil percentage	Laboratory	0.000	✕
Platelet count	Laboratory	0.000	✕
Fibrinogen	Laboratory	0.000	✕
INR	Laboratory	0.000	✕
**Time-related**			

Variable	Category	Coefficient at optimal λ	Selected by LASSO
Onset-to-admission time	Time	0.000	✗
**Etiology (TOAST subtypes)**			✓
LAA vs. SVO	Classification	0.031	
Cardioembolism vs. SVO	Classification	0.083	
Other/undetermined vs. SVO	Classification	0.034	
**Biomarker**			
sCD163	Laboratory	0.215	✓

LASSO parameters:

Candidate predictors were pre-specified based on clinical relevance and prior literature. The pre-specified set included age, sex, hypertension, diabetes, atrial fibrillation, baseline NIHSS, DWI infarct volume, random blood glucose, white blood cell count, neutrophil percentage, platelet count, fibrinogen, INR, onset-to-admission time, TOAST subtypes, and serum sCD163.

Number of candidate variables: 16
Optimal λ (1-SE rule): 0.042
Number of variables with non-zero coefficients: 6 (Age, AF, NIHSS, DWI volume, TOAST , sCD163)

Supplementary Table S6. Full regression coefficients of the final multivariable logistic regression model

Variable	β coefficient	SE	Wald χ^2	aOR	95% CI	P value
sCD163 (high vs. low)	2.015	0.264	58.42	7.52	4.42 – 12.78	<0.001

Variable	β coefficient	SE	Wald χ^2	aOR	95% CI	P value
Baseline NIHSS (per 1 point)	0.298	0.063	22.16	1.35	1.20 – 1.52	<0.001
DWI infarct volume (per 1 mL)	0.057	0.013	18.73	1.06	1.03 – 1.09	<0.001
Atrial fibrillation (yes vs. no)	0.455	0.227	4.02	1.58	1.01 – 2.47	0.045
Constant	-5.123	0.521	96.72	—	—	—

Supplementary Table S7. Missing data patterns in the training and validation cohorts

Variable	Training cohort (n=664)	Validation cohort (n=300)	Total (n=964)
	Missing, n (%)	Missing, n (%)	Missing, n (%)
**Core variables (0% missing)**			
Age	0 (0)	0 (0)	0 (0)
Sex	0 (0)	0 (0)	0 (0)
Atrial fibrillation	0 (0)	0 (0)	0 (0)
Baseline NIHSS	0 (0)	0 (0)	0 (0)
DWI infarct volume	0 (0)	0 (0)	0 (0)

Variable	Training cohort (n=664)	Validation cohort (n=300)	Total (n=964)
TOAST classification	0 (0)	0 (0)	0 (0)
sCD163	0 (0)	0 (0)	0 (0)
3-month mRS	0 (0)	0 (0)	0 (0)
Antiplatelet drug use	0 (0)	0 (0)	0 (0)
Anticoagulant drug use	0 (0)	0 (0)	0 (0)
**Secondary variables**			
Smoking	8 (1.2)	3 (1.0)	11 (1.1)
Alcohol consumption	6 (0.9)	3 (1.0)	9 (0.9)
BMI	10 (1.5)	4 (1.3)	14 (1.5)
Hypertension	3 (0.5)	1 (0.3)	4 (0.4)
Hypercholesterolemia	5 (0.8)	2 (0.7)	7 (0.7)
Diabetes	3 (0.5)	1 (0.3)	4 (0.4)
Family history of stroke	12 (1.8)	5 (1.7)	17 (1.8)
Random blood glucose	32 (4.8)	14 (4.7)	46 (4.8)

Variable	Training cohort (n=664)	Validation cohort (n=300)	Total (n=964)
White blood cell count	24 (3.6)	10 (3.3)	34 (3.5)
Neutrophil percentage	20 (3.0)	8 (2.7)	28 (2.9)
Platelet count	16 (2.4)	7 (2.3)	23 (2.4)
Fibrinogen	28 (4.2)	12 (4.0)	40 (4.1)
INR	14 (2.1)	6 (2.0)	20 (2.1)
**Imaging variables**			
Infarct location	24 (3.6)	10 (3.3)	34 (3.5)
Distribution of culprit vessels	28 (4.2)	12 (4.0)	40 (4.1)
Vessel stenosis degree	24 (3.6)	10 (3.3)	34 (3.5)
Onset-to-admission time	14 (2.1)	6 (2.0)	20 (2.1)
**Total missing**	**271**	**114**	**385**
**Total data points**	**17,928**	**8,100**	**26,028**
**Missing proportion**	**1.51%**	**1.41%**	**1.48%**

Note: Core variables (age, sex, atrial fibrillation, baseline NIHSS, DWI infarct volume, TOAST classification, sCD163, 3-month mRS, antiplatelet drug use, and anticoagulant drug use) had 0% missing data.

Supplementary Table S8. Collinearity diagnostics for pre-specified candidate predictors

Variable	VIF	Tolerance
Age	1.23	0.813
Sex	1.08	0.926
Hypertension	1.14	0.877
Diabetes	1.16	0.862
Atrial fibrillation	1.12	0.893
Baseline NIHSS	1.18	0.847
DWI infarct volume	1.15	0.870
Random blood glucose	1.28	0.781
White blood cell count	1.35	0.741
Neutrophil percentage	1.42	0.704
Platelet count	1.18	0.847
Fibrinogen	1.33	0.752

Variable	VIF	Tolerance
INR	1.19	0.840
Onset-to-admission time	1.09	0.917
TOAST subtypes	1.20	0.833
sCD163	1.21	0.826

VIF (Variance Inflation Factor) quantifies the extent of multicollinearity among predictors. Tolerance is calculated as 1/VIF. VIF < 5 and tolerance > 0.1 are considered acceptable thresholds indicating no significant multicollinearity. All candidate predictors demonstrated VIF values well below 5 (range: 1.08–1.42) and tolerance values above 0.1 (range: 0.704–0.926), confirming the absence of problematic collinearity.

Supplementary Table S9. Interaction effects among independent variables in the final multivariable model

Interaction term	β coefficient	SE	Wald χ^2	P for interaction
sCD163 × NIHSS	0.021	0.018	1.36	0.243
sCD163 × DWI volume	0.008	0.006	1.78	0.182
sCD163 × Atrial fibrillation	0.245	0.198	1.53	0.216
NIHSS × DWI volume	0.012	0.009	1.78	0.182
NIHSS × Atrial fibrillation	0.089	0.075	1.41	0.235

Interaction term	β coefficient	SE	Wald χ^2	P for interaction
DWI volume × Atrial fibrillation	0.032	0.028	1.31	0.252

Note: Interaction terms were introduced into the logistic regression model to test for effect modification between independent variables. A P for interaction > 0.1 indicates no statistically significant interaction. All interaction terms showed P > 0.1, suggesting no substantial interactions among the independent variables in the final model.

Supplementary Table S10. Baseline characteristics of the training and validation cohorts with SMD

Variable	Training cohort (n=664)	Validation cohort (n=300)	SMD	Statistic ($t/\chi^2/Z$)	P value
**Demographic characteristics**					
Age (years, mean±SD)	65.6 ± 11.1	65.3 ± 11.5	**0.027**	t = 0.41	0.682
Male, n (%)	430 (64.8)	192 (64.0)	**0.017**	χ^2 = 0.06	0.810
Smoking, n (%)	253 (38.1)	109 (36.3)	**0.038**	χ^2 = 0.29	0.590
BMI [M (Q1, Q3)]	24.3 (22.6, 26.4)	24.2 (22.5, 26.3)	**0.023**	Z = 0.55	0.582
Alcohol consumption, n (%)	162 (24.4)	78 (26.0)	**0.037**	χ^2 = 0.30	0.584
**Vascular risk factors and comorbidities**					
Hypertension, n (%)	416 (62.7)	191 (63.7)	**0.021**	χ^2 = 0.09	0.765
Hypercholesterolemia, n (%)	273 (41.1)	127 (42.3)	**0.024**	χ^2 = 0.13	0.718

Variable	Training cohort (n=664)	Validation cohort (n=300)	SMD	Statistic ($t/\chi^2/Z$)	P value
Diabetes, n (%)	188 (28.3)	80 (26.7)	**0.036**	$\chi^2 = 0.28$	0.597
Atrial fibrillation, n (%)	167 (25.2)	71 (23.7)	**0.035**	$\chi^2 = 0.25$	0.621
Family history of stroke, n (%)	89 (13.4)	44 (14.7)	**0.038**	$\chi^2 = 0.29$	0.590
**Medication history**					
Antiplatelet drug use, n (%)	302 (45.5)	130 (43.3)	**0.044**	$\chi^2 = 0.40$	0.527
Anticoagulant drug use, n (%)	103 (15.5)	50 (16.7)	**0.032**	$\chi^2 = 0.21$	0.647
**Clinical severity and imaging findings**					
Baseline NIHSS [M (Q1, Q3)]	8 (6, 12)	8 (6, 12)	**0.025**	Z = 0.38	0.704
DWI infarct volume (ml, mean±SD)	19.6 ± 12.5	19.1 ± 12.8	**0.040**	t = 0.57	0.569
Random blood glucose (mmol/L, mean±SD)	8.03 ± 2.82	8.31 ± 2.98	**0.097**	t = 1.38	0.168
White blood cell count ($\times 10^9$/L, mean±SD)	8.40 ± 3.00	8.62 ± 3.18	**0.071**	t = 1.03	0.304
Neutrophil percentage (%, mean±SD)	0.712 ± 0.11	0.728 ± 0.12	**0.139**	t = 1.66	0.097

Variable	Training cohort (n=664)	Validation cohort (n=300)	SMD	Statistic ($t/\chi^2/Z$)	P value
Platelet count ($\times 10^9$/L, mean±SD)	218 ± 56	221 ± 59	**0.052**	t = 0.72	0.471
Fibrinogen (g/L, mean±SD)	3.26 ± 0.79	3.23 ± 0.77	**0.039**	t = 0.54	0.587
INR (mean±SD)	1.03 ± 0.07	1.04 ± 0.08	**0.135**	t = 1.73	0.084
Infarct location, n (%)			**0.073**	χ^2 = 2.21	0.137
– Anterior circulation	379 (57.1)	182 (60.7)			
– Posterior circulation	285 (42.9)	118 (39.3)			
Distribution of culprit vessels, n (%)			**0.087**	χ^2 = 1.89	0.389
– Internal carotid artery	56 (8.4)	21 (7.0)			
– Middle cerebral artery	344 (51.8)	162 (54.0)			
– Basilar artery	264 (39.8)	117 (39.0)			
Vessel stenosis degree, n (%)			**0.085**	χ^2 = 2.99	0.225
– Normal	96 (14.5)	39 (13.0)			
– Stenosis	214 (32.2)	94 (31.3)			

Variable	Training cohort (n=664)	Validation cohort (n=300)	SMD	Statistic ($t/\chi^2/Z$)	P value
– Occlusion	354 (53.3)	167 (55.7)			
Onset-to-admission time (h) [M (Q1, Q3)]	8.0 (4.5, 13.5)	8.0 (4.5, 13.5)	**0.022**	Z = 0.21	0.834
TOAST classification, n (%)			**0.062**	χ^2 = 4.02	0.408
– Large artery atherosclerosis	256 (38.6)	124 (41.3)			
– Cardioembolism	182 (27.4)	87 (29.0)			
– Small vessel occlusion	148 (22.3)	62 (20.7)			
– Other determined etiology	48 (7.2)	14 (4.7)			
– Undetermined etiology	30 (4.5)	13 (4.3)			
**Laboratory biomarker**					
sCD163 (ng/mL, mean±SD)	709 ± 108	705 ± 110	**0.037**	t = 0.54	0.589

Supplementary Table S11 TRIPOD Checklist for Prediction Model Development and Validation

Title and Abstract

Section	Item	Recommendation	Location
**Title**	1a	Identify the study as developing and/or validating a multivariable prediction model	Title page: "Serum sCD163 as a Potential Biomarker for Predicting Poor Functional Outcome at 3 Months in Acute Ischemic Stroke Without Reperfusion: A Prospective Study"
**Title**	1b	Identify the target population, outcome to be predicted, and type of model (prognostic)	Title page (target: AIS patients; outcome: poor functional outcome at 3 months; prognostic model)
**Abstract**	2	Summarize objectives, study design, setting, participants, sample size, predictors, outcome, and model performance	Abstract (lines 1-12)

Introduction

Section	Item	Recommendation	Location
**Background**	3a	Explain the medical context and rationale for developing/validating the model	Introduction, paragraphs 1-3 (lines 1-18)
**Background**	3b	Specify the objectives, including whether the study describes the development, validation, or both	Introduction, last paragraph (lines 19-26)
**Background**	3c	Specify the target population and the intended use of the model	Introduction, last paragraph (lines 19-26)

Methods

Section	Item	Recommendation	Location

Section	Item	Recommendation	Location
**Source of data**	4a	Describe the study design, setting, and data collection period	Methods – Study Population (lines 1-5)
**Source of data**	4b	Specify the key dates of data collection	Methods – Study Population (lines 5-9: January 2021 – December 2024)
**Participants**	5a	Describe the eligibility criteria for participants	Methods – Study Population, Inclusion and Exclusion criteria (lines 15-25)
**Participants**	5b	Describe the diagnostic criteria used	Methods – Study Population (lines 5-7: AHA/ASA diagnostic criteria)
**Outcome**	6	Clearly define the outcome and how it was assessed	Methods – Clinical Data Collection, Outcome definition (mRS at 3 months, blinded assessment, inter-rater reliability)
**Predictors**	7a	Define all predictors used in the model	Methods – Clinical Data Collection / Statistical Analysis (NIHSS, DWI volume, AF, sCD163, age, TOAST)
**Predictors**	7b	Specify how and when predictors were measured	Methods – Clinical Data Collection (NIHSS at admission, DWI within 24h, sCD163 within 24h of admission)
**Sample size**	8	Explain how the study size was determined	Methods – Statistical Analysis (consecutive enrollment, no formal sample size calculation)
**Missing data**	9	Describe how missing data were handled	Methods – Statistical Analysis, Missing data (multiple imputation, mice package, 10 imputations)
**Statistical analysis**	10a	Describe how predictors were selected for the model	Methods – Statistical Analysis, Predictor selection and model development (clinical pre-specification +

Section	Item	Recommendation	Location
			LASSO)
**Statistical analysis**	10b	Describe the model building procedure	Methods – Statistical Analysis (LASSO with 10-fold CV, 1-SE rule → forced entry multivariable logistic regression)
**Statistical analysis**	10c	Describe the type of model	Methods – Statistical Analysis (multivariable logistic regression)
**Statistical analysis**	10d	Specify whether the model was developed for diagnosis or prognosis	Methods – Clinical Data Collection, Outcome definition (prognosis: 3-month functional outcome)
**Statistical analysis**	10e	Describe how the model performance was evaluated	Methods – Statistical Analysis (AUC, calibration plots, HL test, Brier score, DCA)
**Statistical analysis**	10f	Describe how internal validation was performed	Methods – Statistical Analysis, Internal and external validation (bootstrap with 1,000 iterations)
**Statistical analysis**	10g	Describe how external validation was performed	Methods – Statistical Analysis (direct application of original coefficients to independent validation cohort, no recalibration)

Results

Section	Item	Recommendation	Location
**Participants**	11a	Describe the flow of participants through the study	Results – Study population and patient flow / Figure 1

Section	Item	Recommendation	Location
**Participants**	11b	Report the number of participants in each cohort	Results – Study population (training: n=664; validation: n=300)
**Participants**	12a	Describe the baseline characteristics of participants	Results – Table 1 / Supplementary Tables S1-S2
**Participants**	12b	Report the number of events (outcomes) in each cohort	Results – Study population (training: 226 poor outcomes; validation: 102 poor outcomes)
**Model development**	13a	Report the univariable analysis results for all candidate predictors	Results – Table 2 / Figure 3
**Model development**	13b	Report the multivariable analysis results	Results – Table 2 / Figure 4
**Model development**	14a	Present the full prediction model (equation)	Results – Predictor selection ($Z = -5.123 + 2.015 \cdot X_1 + 0.298 \cdot X_2 + 0.057 \cdot X_3 + 0.455 \cdot X_4$)
**Model development**	14b	Present the model coefficients	Supplementary Table S5
**Model performance**	15a	Report the model's discriminative performance (AUC with 95% CI)	Results – Internal Validation (AUC=0.877, 95% CI: 0.829-0.926) and External Validation (AUC=0.867, 95% CI: 0.815-0.921)
**Model performance**	15b	Report the model's calibration performance	Results – Internal Validation (calibration intercept=0.039, slope=0.862, HL P=0.868) and External Validation (calibration intercept=0.032, slope=0.881, HL P=0.680) / Figures 6-7

Section	Item	Recommendation	Location
**Model performance**	16	Report the external validation results	Results – External Validation (AUC=0.867, 95% CI: 0.815-0.921; HL P=0.680)

Discussion

Section	Item	Recommendation	Location
**Discussion**	17	Summarize the key findings and interpret them in context	Discussion, paragraph 1 (lines 1-7)
**Discussion**	18a	Discuss the limitations of the study	Discussion – Limitations (paragraphs 4-5)
**Discussion**	18b	Discuss the potential clinical use of the model	Discussion (throughout: early risk stratification, individualized treatment decisions)
**Discussion**	19	Compare the results with previous studies	Discussion (comparison with Sun et al., Kang et al., Li et al., Yang et al., Liu et al.)
**Discussion**	20	Discuss the implications for future research	Discussion – Conclusion (last paragraph)

Other Information

Section	Item	Recommendation	Location
**Supplementary**	21	Provide the TRIPOD checklist as a supplement	Supplementary Material

Section	Item	Recommendation	Location
**Funding**	22	Report the source of funding and its role	Funding section (Pengcheng Talent - Medical Reserve Talent Fund, 2023)
**Ethics**	23	Report ethical approval and informed consent	Ethics Statement (Approval Nos. XZYY2021-KL01-038 and KZYY2024-KL05-023)
**Data availability**	24	State whether data are available	Data Availability Statement
**Author contributions**	25	Report author contributions	Author Contributions
**Conflict of interest**	26	Report any conflicts of interest	Conflict of Interest (none declared)
